# Tendency of dynamic vasoactive and inotropic medications data as a robust predictor of mortality in patients with septic shock: An analysis of the MIMIC-IV database

**DOI:** 10.3389/fcvm.2023.1126888

**Published:** 2023-03-07

**Authors:** Yi-Le Ning, Ce Sun, Xiang-Hui Xu, Li Li, Yan-Ji Ke, Ye Mai, Xin-Feng Lin, Zhong-Qi Yang, Shao-Xiang Xian, Wei-Tao Chen

**Affiliations:** ^1^Department of Pulmonary and Critical Care Medicine (PCCM), Bao’an District Hospital of Chinese Medicine, Shenzhen, China; ^2^The First Clinical School, Guangzhou University of Chinese Medicine, Guangzhou, China; ^3^Department of Critical Care Medicine, Meizhou Hospital of Chinese Medicine, Meizhou, China; ^4^Department of Critical Care Medicine, Bao’an District Hospital of Chinese Medicine, Shenzhen, China; ^5^Department of Pulmonary and Critical Care Medicine (PCCM), The First People’s Hospital of Kashgar Prefecture, Kashgar, China; ^6^Department of Critical Care Medicine, The Fourth People’s Hospital of Foshan, Foshan, China; ^7^Department of Critical Care Medicine, Chinese Medicine Hospital of Hainan Province, Haikou, China; ^8^Department of Critical Care Medicine, The First Affiliated Hospital of Guangzhou University of Chinese Medicine, Guangzhou, China; ^9^The First Affiliated Hospital of Guangzhou University of Chinese Medicine, Guangzhou, China

**Keywords:** vasoactive-inotropic score, mortality, septic shock, high-resolution data, VIS reduction rate, critical care

## Abstract

**Background:**

Septic shock patients fundamentally require delicate vasoactive and inotropic agent administration, which could be quantitatively and objectively evaluated by the vasoactive–inotropic score (VIS); however, whether the dynamic trends of high-time-resolution VIS alter the clinical outcomes remains unclear. Thus, this study proposes the term VIS Reduction Rate (VRR) to generalise the tendency of dynamic VIS, to explore the association of VRR and mortality for patients with septic shock.

**Methods:**

We applied dynamic and static VIS data to predict ICU mortality by two models: the long short-term memory (LSTM) deep learning model, and the extreme gradient boosting (XGBoost), respectively. The specific target cohort was extracted from the Medical Information Mart for Intensive Care IV (MIMIC-IV) database by the sophisticated structured query language (SQL). Enrolled patients were divided into four groups by VRR value: ≥50%, 0 ~ 50%, −50% ~ 0, and < −50%. Statistical approaches included pairwise propensity score matching (PSM), Cox proportional hazards regression, and two doubly robust estimation models to ensure the robustness of the results. The primary and secondary outcomes were ICU mortality and in-hospital mortality, respectively.

**Results:**

VRR simplifies the dosing trends of vasoactive and inotropic agents represented by dynamic VIS data while requiring fewer data. In total, 8,887 septic shock patients were included. Compared with the VRR ≥50% group, the 0 ~ 50%, −50% ~ 0, and < −50% groups had significantly higher ICU mortality [hazard ratio (HR) 1.32, 95% confidence interval (CI) 1.17–1.50, *p* < 0.001; HR 1.79, 95% CI 1.44–2.22, *p* < 0.001; HR 2.07, 95% CI 1.61–2.66, *p* < 0.001, respectively] and in-hospital mortality [HR 1.43, 95% CI 1.28–1.60, p < 0.001; HR 1.75, 95% CI 1.45–2.11, *p* < 0.001; HR 2.00, 95% CI 1.61–2.49, *p* < 0.001, respectively]. Similar findings were observed in two doubly robust estimation models.

**Conclusion:**

The trends of dynamic VIS in ICU might help intensivists to stratify the prognosis of adult patients with septic shock. A lower decline of VIS was remarkably associated with higher ICU and in-hospital mortality among septic shock patients receiving vasoactive–inotropic therapy for more than 24 h.

## Background

Hemodynamic instability is one of the most common characteristics of sepsis in clinical practice (1). Specifically, absolute or relative insufficiency of blood volume due to systemic vasodilation leads to persistent hypotension (2). Simultaneously with early adequate volume resuscitation and antibiotic therapy, vasoactive–inotropic treatment is a fundamental clinical intervention for septic shock, which aims to maintain a mean arterial pressure of at least 65 mmHg to ensure normal tissue perfusion and hemodynamic stability (3–5). The temporal trends in the utilization of vasoactive–inotropic agents, including the real-time dose rate and duration, reflect severity in patients with septic shock (6).

Although vasoactive–inotropic agents have been widely used clinically in the treatment of sepsis and septic shock, it is recognized that excessive doses of vasoactive medications may be associated with several adverse events, such as arrhythmias, myocardial injury, and tissue hypoperfusion due to excessive vasoconstriction (7–9). Few studies focused on the temporal trends of vasoactive agents’ dosage and mortality (10), and we found no publications that specifically reported how the tendency of these multiple vasoactive agents’ dosage influence mortality in patients with septic shock. Therefore, the relationship between the reduction in utilization of multiple vasoactive–inotropic agents and septic mortality needs further evaluation.

Multiple vasoactive–inotropic agents are applied to stabilize the hemodynamic condition of septic shock, including norepinephrine, epinephrine, dopamine, dobutamine, and vasopressin, with enormous heterogeneity in clinical practice (6, 11, 12). The vasoactive–inotropic score (VIS) is a tool widely deployed to assess the dosage of vasoactive agents quantitatively and was proposed by Gaies in 2010 (13). In pediatric sepsis, the VIS is independently associated with clinical outcomes such as mortality, duration of mechanical ventilation, and ICU length of stay and can be used as an early predictor (14, 15). Similarly, in cardiac surgery and heart transplantation, the increase in VIS can also be used as a predictor of postoperative mortality, suggesting that the increase in the use of vasoactive–inotropic agents is related to the increase in mortality (13, 16–20). However, the relationship between the clinical outcome of adult patients with septic shock and temporal change in VIS has not been studied.

Similar to other continuous data in ICU, the VIS appears as high-resolution data that changes over time. These 1 h resolution data can provide real-time information for judging the patient’s status, and they can be used to estimate the prognosis. Machine learning and deep learning algorithms have unique advantages in processing high-resolution data. An increasing number of studies have found that machine learning and deep learning models perform better in high-resolution data to predict disease prognosis than traditional methods (21–23).

This study aimed to describe the correlation between a reduction in early vasoactive–inotropic score and mortality in septic shock. To verify whether VIS data can predict ICU mortality alone, we constructed a long short-term memory (LSTM) deep learning model and an extreme gradient boosting (XGBoost) machine learning model with dynamic and static VIS data, respectively. We developed the term VIS reduction rate (VRR) to evaluate the decrease in vasoactive agents quantitatively. Using this definition, we illustrated the relationship between a reduction in vasoactive medications within 48 h and ICU mortality in patients with septic shock.

## Methods

### Study cohort

The target cohort in this study was obtained from the Medical Information Mart for Intensive Care IV (MIMIC-IV) version 2.0 (24), which is a freely accessible critical care database that contains detailed demographics, laboratory tests, various notes and reports, and real-time high-resolution hourly data (vital signs, intravenous medications infusion rates, etc.) acquired from the hardware devices (monitor, infusion pumps, etc.) of over 76,000 patients during ICU admission at Beth Israel Deaconess Medical Center. This study aimed to determine whether VRR is significantly associated with ICU mortality and in-hospital mortality for patients with septic shock. Briefly, we utilized the following inclusion criteria in the identification of the study cohort: (1) patients with sepsis defined by Sepsis-3 criteria; (2) length of stay in ICU over 24 h; (3) age 18 years or older; (4) weight data (if needed) to calculate particular subsections of the VIS; (5) treatment with at least one vasoactive–inotropic agent; (6) therapy with vasoactive–inotropic agents within 24 h after ICU admission; and (7) subsequent ICU stay greater than 24 h after initiation of vasoactive–inotropic therapy. Exclusion criteria: discharge from ICU against medical advice.

### Data extraction

The real-time VIS was queried and calculated from the immediate infusion rates of dopamine, dobutamine, epinephrine, milrinone, vasopressin, and norepinephrine. For multiple infusion rates recorded in a given hour for the same vasoactive–inotropic agent, we took the maximum rate as the input value in the specific hour for the dynamic VIS data. The dynamic VIS data with 1 h temporal resolution of patients contained 7 features: 6 subsections of VIS (VIS of dopamine, dobutamine, epinephrine, milrinone, vasopressin, norepinephrine) and the total VIS.

Then, we took the maximum value of each VIS item within 1–24 and 25–48 h and calculated VRR, thus forming a data matrix with 15 features (maximum VIS of dopamine, dobutamine, epinephrine, milrinone, vasopressin, norepinephrine, total VIS within 1–24 and 25–48 h, respectively, and VRR). We called the data matrix with 15 features “static VIS data” by comparison with the high-resolution dynamic VIS data.

Only the data during the first 24 h for each patient after ICU admission were used as covariates in this study for further analysis. We merged the fields of demographic and admission information, severity scores, comorbidities, vital signs, interventions, laboratory tests, VRR, and outcome to form the final cohort data matrix.

### VIS and VRR

The formula of VIS is defined as follows (13): VIS = dopamine dose (μg·kg^−1^·min^−1^) + dobutamine dose (μg·kg^−1^·min^−1^) + 100 × epinephrine dose (μg·kg^−1^·min^−1^) + 10 × milrinone dose (μg·kg^−1^·min^−1^) + 100 × norepinephrine dose (μg·kg^−1^·min^−1^) + 10,000 × vasopressin dose (U·kg^−1^·min^−1^).

VRR is a totally new concept that we proposed to quantify dosage reduction for vasoactive–inotropic agents. The formula of VRR is defined as follows: (VIS_1-24h max_ – VIS_25-48h max_)/VIS_1-24h max_.

### Outcome

The primary outcome in this study was ICU mortality, and the secondary outcome was in-hospital mortality.

### Deep learning and machine learning model with VIS data

**Figure 1:**
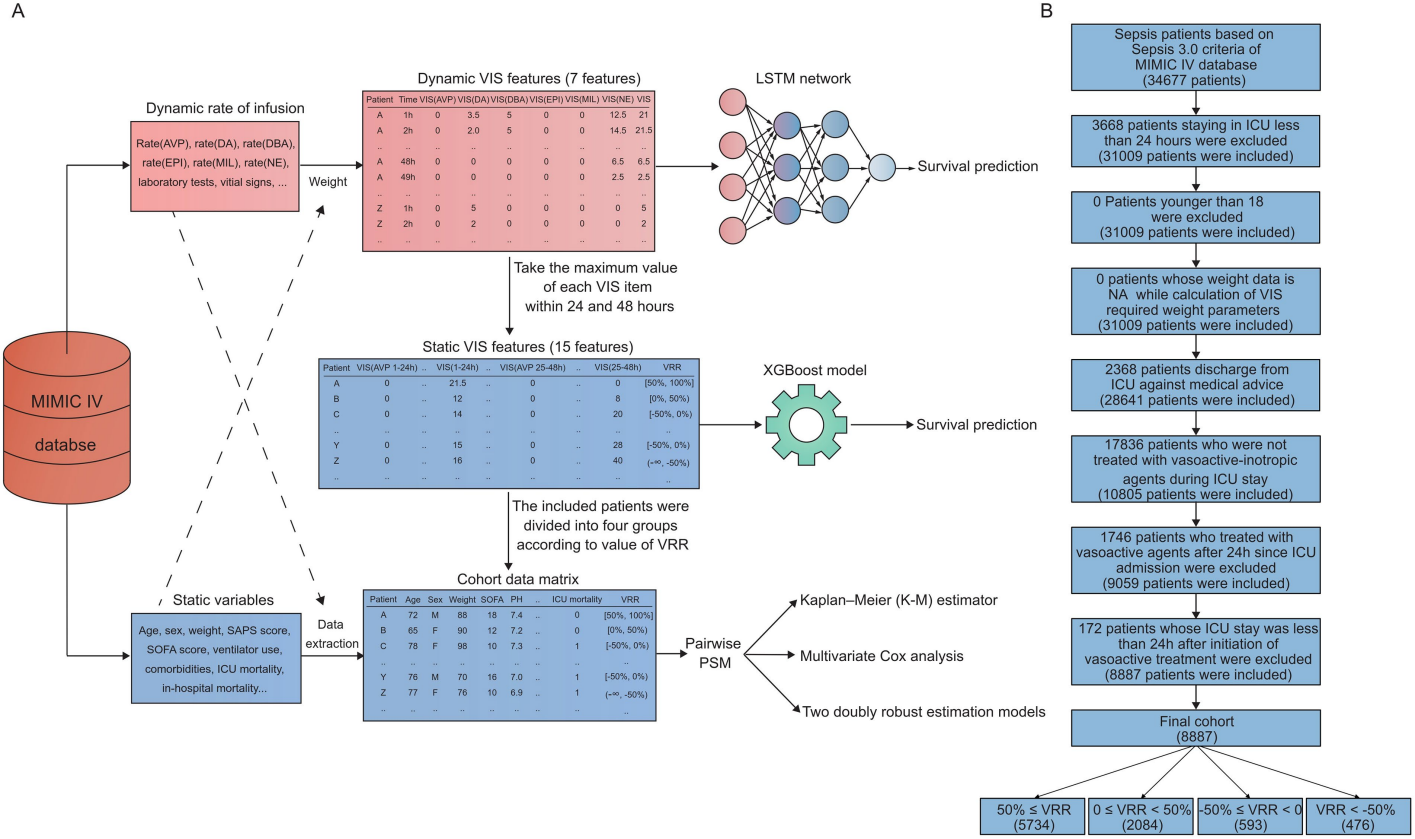
Schematic illustration and flow chart. **(A)** Raw data were obtained from the MIMIC-IV databases. A survival prediction model was trained using an LSTM network, which was updated hourly. Static VIS data were characterized by 15 features, including VRR and VIS features at 1–24 h and 25–48 h, respectively, which were extracted based on the dynamic VIS data. Then, according to the results of machine learning, we selected the most representative feature, VRR, for further statistical analysis. LSTM, long short-term memory; VIS, vasoactive–inotropic score; VRR, VIS reduction rate; AVP, vasopressin; DA, dopamine; DBA, dobutamine; EPI, epinephrine; MIL, milrinone; NE, norepinephrine. **(B)** The study flow diagram.

The data were randomly split into a training dataset (80%) and a test dataset (20%) according to the labels of outcome events by the caTools package in R version 4.0.2. Constant “stay_id” in the training data and validation datasets were used in the deep learning model with dynamic data and the machine learning model with static data. A schematic illustration of this study is shown in Figure 1A.

The LSTM is a special modified recurrent neural network (RNN) that is widely used for the prediction of time series data in ICU due to the property of handling previous information for a relatively long period of time. Based on the 1 h high-resolution characteristics of ICU data, the prediction performance of LSTM has been fully developed over time, making it one of the most state-of-the-art deep learning algorithms for dynamic data in ICU. In this study, we processed the dynamic VIS data with the LSTM model with PyTorch version 1.8.0 accelerated by NVIDIA GeForce RTX 2060 SUPER graphics processing units (ZOTAC, Hong Kong). We set 200, 64, 0.0001, 0.25 as parameter “epochs,” “batch size,” “learning rate,” “dropout” for LSTM model, respectively. All the subjects of time series data needed the same length to train the model in batches; however, enrolled patients may have had inconsistent ICU stays within 48 h after initiating vasoactive–inotropic therapy. We referred to Thorsen-Meyer et al.’s method for processing dynamic time series data (21), and patients who did not match the given time point (1–48 h) were removed to generate training data with the same length.

The XGBoost is an optimized version of gradient boosted decision trees to implement classification and regression predictive modeling problems (25). The XGBoost algorithm made the following improvements under the gradient boosting framework: regularized boosting function to avoid overfitting, handling of missing values automatically, cross-validation at each iteration, incremental training, and tree pruning. Based on the performance and scalability mentioned above, the XGBoost algorithm is becoming increasingly popular in big data for critical care. We utilized Python version 3.8.8 to train an XGBoost machine learning model to predict ICU mortality with a total of 15 features, including the VRR and static maximum data mentioned above. We set ‘logloss’ as parameter “eval_metric” for XGBoost model. We applied the SHapley Additive exPlanations (SHAP) algorithm (26) in our XGBoost model to obtain explanations of the features that dominate ICU mortality predictions of patients with septic shock.

### Statistical analysis

We divided patients with septic shock into 4 groups according to the VRR value: 50% ≤ VRR, 0 ≤ VRR < 50%, −50% ≤ VRR < 0, and VRR < −50%. Univariate Cox analysis was first performed to identify candidate variables that were considered to be clinically relevant for further multivariate Cox analysis, with a value of *p* < 0.05 as the cut-off value. We performed survival analysis with the Kaplan–Meier estimator and multivariate Cox proportional hazards regression analysis among these 4 groups. To further control the influence of confounding factors, we also conducted pairwise propensity score matching (PSM) (27, 28) among these 4 groups to handle the missing data and avoid bias. We set 500 and “pmm” as parameter “seed” and “method” in mice function for multiple imputation with mice package in R before PSM for pairwise primary cohorts. PSM was performed with the MatchIt package, and a total of 6 matched cohorts were generated.

We performed the Anderson–Darling normality test to determine whether the data were normally distributed and Bartlett’s test (original cohort, 4 groups) or F test (pairwise cohort, 2 groups) to assess the equality of variances. If the data among groups followed normal distributions and the variances to be compared were homogeneous, we utilized one-way ANOVA (original cohort, 4 groups) or *t*-test (pairwise cohort, 2 groups) to test the differences for continuous covariates; otherwise, Kruskal–Wallis test (original cohort, 4 groups) or Wilcoxon test (pairwise cohort, 2 groups) was applied as appropriate. The Chi-square test was used to test the differences for categorical covariates. All the statistical approaches mentioned above were executed in R version 4.0.2.

### Sensitivity analysis

To obtain a robust conclusion, for these 6 matched cohorts, we applied a series of sensitivity analyses including the Kaplan–Meier (K-M) estimator, multivariate Cox regression analysis, and two doubly robust estimation models: the survey-weighted generalized linear model with all covariates using the inverse probability-weighting (IPW) technique calculated from PSM and the survey-weighted Cox model with all covariates using IPW for ICU mortality and in-hospital mortality separately.

### Covariates

We referred to a previous well-designed research (29) on sepsis to define the covariates in our study: (1) demographic information(age, gender, weight); (2) severity scores including simplified acute physiology score (SAPS) II, sequential organ failure assessment (SOFA) score, first nonzero VIS; (3) comorbidities including atrial fibrillation (AFIB), coronary artery disease (CAD); Congestive heart failure (CHF), chronic obstructive pulmonary disease (COPD), liver disease, malignant tumor and chronic renal disease; (4) vital signs including heart rate, mean arterial pressure (MAP), temperature; (5) interventions including mechanical ventilation, sedative therapy; and (6) laboratory tests including white blood cell (WBC) count, hemoglobin, platelet count, sodium, potassium, bicarbonate, partial pressure of oxygen (PO2), partial pressure of carbon dioxide(PCO2); central venous pressure (CVP), etc.

## Results

A total of 34,677 patients were identified as having sepsis by the Sepsis-3 criteria. To perform a precise definition of patients who were transferred to ICU due to septic shock, we included the initiation of vasoactive agents therapy within 24 h after ICU admission as one of the inclusion criteria. Furthermore, to ensure VIS data for 1–24 and 25–48 h were available, we limited the subsequent ICU stay of included patients to more than 24 h after treatment with vasoactive agents. In addition, patients who were on the mend were normally transferred from ICU to the general ward as a transition. To avoid bias in ICU mortality and in-hospital mortality of discharge contributed to by nonmedical factors such as medical insurance, we defined subjects with the same ICU transfer time and discharge time as patients who were discharged from ICU against medical advice and excluded them from the target cohort. The specific inclusion and exclusion process is shown in a flow chart (Figure 1B). Ultimately, we acquired a final cohort of 8,887 patients.

### Association between VIS data and ICU mortality

To clarify whether early VIS data could predict patient prognosis, we extracted the first 48 h VIS data after initiating vasoactive–inotropic therapy into dynamic and static VIS data. As the schematic illustration shows (Figure 1A), dynamic VIS data are a real-time, 1 h-resolution data matrix containing 7 features: the VIS of dopamine, dobutamine, epinephrine, milrinone, vasopressin, and norepinephrine, and the total VIS. Static VIS data were characterized with 15 features, including VRR and VIS features at 1–24 h and 25–48 h, respectively, which were extracted based on the dynamic VIS data.

**Figure 2:**
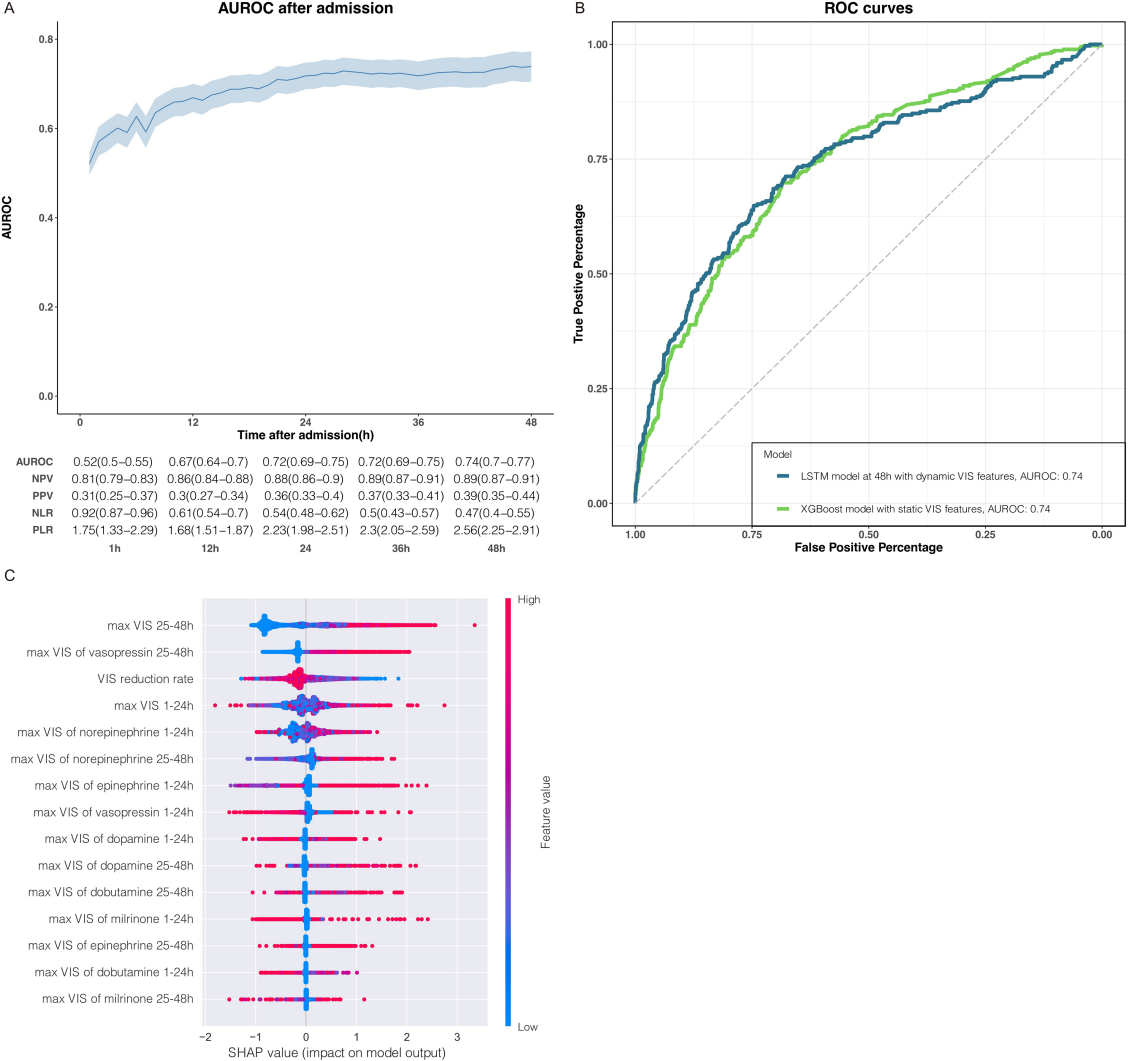
Association between VIS data and ICU mortality. **(A)** The performance of LSTM deep learning models were trained with dynamic VIS data during 0–1 h, 1–2 h, 1–3 h, 1–4 h, 1–5 h, 1–6 h and so on, constant increase for 1 h until 1–48 h. Model performance increasingly improved with the accumulation of the dynamic VIS data after ICU admission. The metrics for each timepoint in the graphs are displayed in the tables below with a 95% CI in parentheses. AUROC, area under the receiver operating characteristic; NPV, negative predictive value; PPV, positive predictive value; NLR, negative likelihood ratio; PLR, positive likelihood ratio. **(B)** Receiver operating characteristic (ROC) curves of the LSTM model and XGBoost model trained with the dynamic and the static VIS data, respectively. **(C)** The impact of the static VIS features on predictions.

We applied dynamic and static VIS data to train the LSTM deep learning model and XGBoost machine learning model to predict ICU mortality, respectively. The LSTM model was trained with temporal data, which was capable of updating the prediction of ICU mortality based on learning hourly VIS characteristics accumulated over time. Unsurprisingly, the prediction accuracy of mortality by the LSTM model trained with 7 VIS features was increasingly improved to some extent with the extension of the time dimension of the VIS data (Figure 2A). In addition, the improvement of prediction accuracy has reached a bottleneck to a certain extent over time, which may be related to too few features being included. Compared with the LSTM model, which requires extensive and high-resolution temporal data, the XGBoost model trained with less static VIS data had a similar model performance for the prediction of ICU mortality (Figure 2B). VRR simplifies the dosing trends of vasoactive and inotropic agents represented by dynamic VIS data while using less data. Although we converted dynamic data to static data, VRR represented a significant relationship with patient mortality in terms of model predictive performance. Since the VRR is calculated by the VIS data in two consecutive time periods, it still represents the dynamic trend of the dose of vasoactive drugs to some extent. Through the prediction model established by our work above, we were able to predict ICU mortality to some extent by relying solely on VIS data, which led us to wonder which VIS feature made an essential contribution to the predictive output of the model. Next, we applied the SHAP algorithm quantitatively and visually explained the XGBoost model (Figure 2C).

The SHAP summary plot displayed how the top features of VIS data impact our XGBoost model output for ICU mortality. Order on the y-axis represented the magnitude of the average impact of VIS features on our model (Figure 2C; Supplementary Figure 1). The colorful dots from blue to red indicated the value of continuous VIS data from low to high (Figure 2C), and the position on the left or right side of the x-axis implied whether the prediction toward ICU survival (right side: favor to ICU survival, left side: favor to ICU non-survival). The top 6 impactful VIS features of the XGBoost model for ICU mortality were VIS_25-48h max_, VIS_25-48h max_ of vasopressin, VRR, VIS_1-24h max_ of norepinephrine, and VIS_25-48h max_ of norepinephrine.

Unsurprisingly, VIS_25-48h max_ had the most significant role in our trained model, implying that the higher the VIS during 25–48 h is, the higher the tendency toward non-survival. However, the contribution of VIS_1-24h max_ to the model output was slightly more complex but also made sense: there are some individuals on the left side with higher VIS_1-24h max_ values, but the prediction still favors survival, and a similar paradox exists on the right side of VIS_1-24h max_. The seemingly contradictory predictions can be explained by some clinically common phenomena, such as patients in ICU with higher VIS at the beginning of ICU admission but whose hemodynamics improve the next day as the treatment progresses so that the dose rate of their vasoactive agents is markedly reduced. Therefore, the dynamic change in VIS highlights the predictive role in the model; it is hardly accidental that the higher the VRR is, the greater the tendency toward survival in ICU will be. The important role of VIS (norepinephrine) at 1–24 h and 25–48 h in the model was also confirmed: norepinephrine is the first choice in hemodynamic support therapy for patients with septic shock, and the effects of the VIS (norepinephrine) were similar to those of VIS_25-48h max_ and VIS_1-24h max_. In addition to norepinephrine, the max dose rate of vasopressin during 25–48 h also had impactful features of model output, which conformed to the recommendations for vasoactive agents in the latest Surviving Sepsis Campaign Guidelines from 2021 on the one hand, but also indicates that a large dose of vasopressin in late stage is not conducive to the prognosis of ICU outcome.

In general, we successfully illustrated a significant association between VIS data and ICU mortality with a machine learning algorithm with small amounts of static VIS data and a deep learning algorithm with large amounts of dynamic data. We quantified and visualized the dynamic change in VIS data represented by VRR, which is an important indicator of patient prognosis in ICU.

### Study cohort and patient characteristics

In view of VIS unified 6 widely used vasoactive agents in ICU under one metric, and its spatiotemporal dynamic change was significantly associated with the prognosis of ICU patients, we divided the patients of the cohort into four groups according to the VRR value (Figure 1B): 50% ≤ VRR (*n* = 5,743), 0 ≤ VRR < 50% (*n* = 2084), −50% ≤ VRR < 0 (*n* = 593), and VRR < −50% (*n* = 476). The basic demographic characteristics of the original cohort are shown in Supplementary Table 1. As shown, the baseline values of some pivotal variables, such as the SAPS II, SOFA score, and first nonzero VIS were significantly imbalanced. Then, we combined the four cohorts for each pairwise comparison, resulting in six new sets of cohorts (cohorts 1–6). To avoid baseline imbalances between groups and the bias caused by missing data, we performed multiple imputation for missing data and matched the imputed cohorts with 1:1 pairwise PSM. Table 1 shows the baseline characteristics of the six cohorts after 1:1 pairwise PSM: cohort 1 (50% ≤ VRR versus 0 ≤ VRR < 50%, 2046 pairs), cohort 2 (50% ≤ VRR versus −50% ≤ VRR < 0, 592 pairs), cohort 3 (50% ≤ VRR versus VRR < − 50%, 475 pairs), cohort 4 (0 ≤ VRR < 50% versus −50% ≤ VRR < 0, 592 pairs), cohort 5 (0 ≤ VRR < 50% versus VRR < −50%, 473 pairs), and cohort 6 (−50% ≤ VRR < 0 versus VRR < −50%, 452 pairs). The covariate characteristics of each cohort before and after PSM are shown in Supplementary Tables 2–7. As shown there, the covariates with standardized mean differences (SMDs) less than 0.1 in the most matched cohorts increased significantly compared with those before matching, indicating that the baseline of the matched cohort was better balanced.

**Table 1:** Basic demographic of matched 6 cohorts after multiple imputation.

	Cohort 1		Cohort 2		Cohort 3		Cohort 4		Cohort 5		Cohort 6	
	50% ≤ VRR (*N* = 2,046)	0 ≤ VRR < 50% (*N* = 2,046)	50% ≤ VRR (*N* = 592)	−50% ≤ VRR < 0 (*N* = 592)	50% ≤ VRR (*N* = 475)	VRR < −50% (*N* = 475)	0 ≤ VRR < 50% (*N* = 592)	−50% ≤ VRR < 0 (*N* = 592)	0 ≤ VRR < 50% (*N* = 473)	VRR < −50% (*N* = 473)	−50% ≤ VRR < 0 (*N* = 452)	VRR < −50% (*N* = 452)
Age												
Mean (SD)	67.10 (15.06)	67.01 (15.17)	66.99 (14.78)	66.43 (14.95)	66.91 (14.51)	66.63 (14.63)	66.42 (15.72)	66.45 (14.94)	66.89 (15.79)	66.72 (14.57)	66.83 (14.70)	66.69 (14.56)
Gender												
Female	888 (43.40%)	868 (42.42%)	270 (45.61%)	254 (42.91%)	212 (44.63%)	199 (41.89%)	255 (43.07%)	254 (42.91%)	202 (42.71%)	198 (41.86%)	196 (43.36%)	189 (41.81%)
Male	1,158 (56.60%)	1,178 (57.58%)	322 (54.39%)	338 (57.09%)	263 (55.37%)	276 (58.11%)	337 (56.93%)	338 (57.09%)	271 (57.29%)	275 (58.14%)	256 (56.64%)	263 (58.19%)
Weight												
Mean (SD)	83.34 (24.17)	83.30 (24.87)	82.92 (24.80)	83.09 (25.21)	83.28 (24.60)	83.25 (26.18)	83.31 (24.30)	83.14 (25.24)	82.92 (23.92)	83.40 (26.19)	82.19 (22.35)	83.16 (26.17)
SAPS II												
Mean (SD)	49.40 (14.68)	49.58 (15.01)	51.37 (15.85)	51.71 (14.82)	49.29 (15.59)	49.56 (14.62)	50.56 (16.22)	51.77 (14.90)	48.55 (15.48)	49.62 (14.81)	50.05 (14.54)	50.12 (14.86)
SOFA score												
Mean (SD)	10.78 (3.64)	10.82 (3.67)	11.48 (3.70)	11.64 (3.91)	11.05 (3.82)	11.11 (3.76)	11.43 (3.97)	11.65 (3.93)	10.88 (3.88)	11.09 (3.76)	11.10 (3.73)	11.22 (3.81)
First nonzero VIS												
Mean (SD)	15.80 (17.21)	15.61 (15.15)	13.59 (13.11)	13.18 (12.23)	11.67 (12.67)	10.73 (11.93)	12.45 (11.75)	13.15 (12.24)	10.71 (11.08)	10.48 (10.59)	11.22 (9.87)	10.70 (10.76)
Mechanical ventilation												
NO	828 (40.47%)	827 (40.42%)	235 (39.70%)	232 (39.19%)	219 (46.11%)	217 (45.68%)	250 (42.23%)	233 (39.36%)	235 (49.68%)	215 (45.45%)	199 (44.03%)	201 (44.47%)
YES	1,218 (59.53%)	1,219 (59.58%)	357 (60.30%)	360 (60.81%)	256 (53.89%)	258 (54.32%)	342 (57.77%)	359 (60.64%)	238 (50.32%)	258 (54.55%)	253 (55.97%)	251 (55.53%)
Sedative												
NO	549 (26.83%)	558 (27.27%)	139 (23.48%)	148 (25.00%)	135 (28.42%)	145 (30.53%)	154 (26.01%)	148 (25.00%)	150 (31.71%)	145 (30.66%)	136 (30.09%)	134 (29.65%)
YES	1,497 (73.17%)	1,488 (72.73%)	453 (76.52%)	444 (75.00%)	340 (71.58%)	330 (69.47%)	438 (73.99%)	444 (75.00%)	323 (68.29%)	328 (69.34%)	316 (69.91%)	318 (70.35%)
AFIB												
NO	1,641 (80.21%)	1,644 (80.35%)	477 (80.57%)	474 (80.07%)	376 (79.16%)	368 (77.47%)	480 (81.08%)	474 (80.07%)	369 (78.01%)	365 (77.17%)	351 (77.65%)	352 (77.88%)
YES	405 (19.79%)	402 (19.65%)	115 (19.43%)	118 (19.93%)	99 (20.84%)	107 (22.53%)	112 (18.92%)	118 (19.93%)	104 (21.99%)	108 (22.83%)	101 (22.35%)	100 (22.12%)
CAD												
NO	1,346 (65.79%)	1,340 (65.49%)	371 (62.67%)	379 (64.02%)	285 (60.00%)	295 (62.11%)	382 (64.53%)	379 (64.02%)	278 (58.77%)	293 (61.95%)	276 (61.06%)	282 (62.39%)
YES	700 (34.21%)	706 (34.51%)	221 (37.33%)	213 (35.98%)	190 (40.00%)	180 (37.89%)	210 (35.47%)	213 (35.98%)	195 (41.23%)	180 (38.05%)	176 (38.94%)	170 (37.61%)
CHF												
NO	1,169 (57.14%)	1,190 (58.16%)	351 (59.29%)	361 (60.98%)	271 (57.05%)	274 (57.68%)	345 (58.28%)	361 (60.98%)	260 (54.97%)	273 (57.72%)	264 (58.41%)	263 (58.19%)
YES	877 (42.86%)	856 (41.84%)	241 (40.71%)	231 (39.02%)	204 (42.95%)	201 (42.32%)	247 (41.72%)	231 (39.02%)	213 (45.03%)	200 (42.28%)	188 (41.59%)	189 (41.81%)
COPD												
NO	1707 (83.43%)	1705 (83.33%)	485 (81.93%)	476 (80.41%)	403 (84.84%)	393 (82.74%)	466 (78.72%)	476 (80.41%)	389 (82.24%)	391 (82.66%)	377 (83.41%)	371 (82.08%)
YES	339 (16.57%)	341 (16.67%)	107 (18.07%)	116 (19.59%)	72 (15.16%)	82 (17.26%)	126 (21.28%)	116 (19.59%)	84 (17.76%)	82 (17.34%)	75 (16.59%)	81 (17.92%)
Liver												
NO	1804 (88.17%)	1807 (88.32%)	513 (86.66%)	509 (85.98%)	419 (88.21%)	419 (88.21%)	493 (83.28%)	510 (86.15%)	404 (85.41%)	417 (88.16%)	404 (89.38%)	396 (87.61%)
YES	242 (11.83%)	239 (11.68%)	79 (13.34%)	83 (14.02%)	56 (11.79%)	56 (11.79%)	99 (16.72%)	82 (13.85%)	69 (14.59%)	56 (11.84%)	48 (10.62%)	56 (12.39%)
Malignancy												
NO	1,655 (80.89%)	1,647 (80.50%)	477 (80.57%)	477 (80.57%)	385 (81.05%)	379 (79.79%)	482 (81.42%)	477 (80.57%)	390 (82.45%)	377 (79.70%)	364 (80.53%)	358 (79.20%)
YES	391 (19.11%)	399 (19.50%)	115 (19.43%)	115 (19.43%)	90 (18.95%)	96 (20.21%)	110 (18.58%)	115 (19.43%)	83 (17.55%)	96 (20.30%)	88 (19.47%)	94 (20.80%)
Renal												
NO	1,379 (67.40%)	1,402 (68.52%)	368 (62.16%)	396 (66.89%)	290 (61.05%)	312 (65.68%)	404 (68.24%)	396 (66.89%)	314 (66.38%)	311 (65.75%)	300 (66.37%)	298 (65.93%)
YES	667 (32.60%)	644 (31.48%)	224 (37.84%)	196 (33.11%)	185 (38.95%)	163 (34.32%)	188 (31.76%)	196 (33.11%)	159 (33.62%)	162 (34.25%)	152 (33.63%)	154 (34.07%)
Stroke												
NO	1889 (92.33%)	1885 (92.13%)	551 (93.07%)	546 (92.23%)	429 (90.32%)	428 (90.11%)	540 (91.22%)	546 (92.23%)	419 (88.58%)	426 (90.06%)	410 (90.71%)	410 (90.71%)
YES	157 (7.67%)	161 (7.87%)	41 (6.93%)	46 (7.77%)	46 (9.68%)	47 (9.89%)	52 (8.78%)	46 (7.77%)	54 (11.42%)	47 (9.94%)	42 (9.29%)	42 (9.29%)
Heart rate												
Mean (SD)	94.11 (22.25)	94.18 (21.58)	93.15 (22.16)	93.64 (20.70)	92.69 (22.02)	93.01 (21.09)	92.10 (22.08)	93.66 (20.70)	90.29 (21.70)	92.94 (21.07)	92.70 (20.82)	92.81 (21.33)
MAP												
Mean (SD)	76.56 (18.73)	76.46 (19.27)	76.51 (18.61)	75.77 (19.10)	77.69 (19.62)	77.56 (19.32)	74.89 (18.50)	75.82 (19.23)	76.34 (19.73)	77.39 (19.18)	77.16 (19.63)	77.22 (18.89)
Temperature												
Mean (SD)	36.58 (1.10)	36.61 (1.13)	36.45 (1.21)	36.52 (1.29)	36.51 (1.11)	36.55 (1.12)	36.50 (1.20)	36.51 (1.30)	36.53 (1.16)	36.55 (1.12)	36.52 (1.22)	36.54 (1.20)
WBC												
Mean (SD)	15.14 (9.92)	14.95 (10.68)	14.98 (9.31)	14.77 (9.28)	14.58 (8.89)	14.70 (8.90)	14.26 (12.20)	14.78 (9.27)	14.32 (13.07)	14.78 (8.93)	14.86 (9.34)	14.77 (8.95)
Hemoglobin												
Mean (SD)	10.28 (2.40)	10.26 (2.32)	10.16 (2.40)	10.25 (2.23)	10.13 (2.36)	10.26 (2.36)	10.17 (2.32)	10.24 (2.23)	10.23 (2.35)	10.27 (2.38)	10.29 (2.18)	10.27 (2.38)
Platelet												
Mean (SD)	201.98 (123.26)	203.76 (123.74)	192.17 (127.70)	191.41 (120.29)	191.14 (123.64)	188.64 (105.97)	188.93 (125.11)	191.18 (120.45)	178.27 (103.43)	189.30 (106.26)	189.33 (115.72)	188.77 (107.77)
Sodium												
Mean (SD)	137.01 (6.12)	136.96 (5.93)	136.90 (6.28)	137.09 (6.17)	136.59 (6.09)	136.61 (5.82)	137.07 (6.35)	137.06 (6.17)	136.66 (6.00)	136.63 (5.80)	136.69 (6.07)	136.68 (5.87)
Potassium												
Mean (SD)	4.33 (0.91)	4.32 (0.91)	4.42 (0.94)	4.44 (0.92)	4.35 (0.88)	4.38 (0.90)	4.43 (0.93)	4.43 (0.92)	4.36 (0.89)	4.37 (0.90)	4.37 (0.85)	4.39 (0.88)
Chloride												
Mean (SD)	103.22 (7.64)	103.08 (7.72)	103.09 (8.09)	102.85 (7.99)	102.65 (7.97)	102.50 (8.08)	103.02 (8.11)	102.85 (7.98)	102.83 (7.91)	102.49 (8.07)	102.60 (8.01)	102.51 (8.05)
Bun												
Mean (SD)	37.42 (27.32)	37.12 (26.53)	41.72 (31.10)	40.21 (28.69)	41.60 (31.89)	39.96 (28.92)	40.36 (30.02)	40.29 (28.67)	40.25 (29.35)	40.29 (29.56)	41.20 (30.10)	40.08 (29.40)
Creatinine												
Mean (SD)	2.07 (1.84)	2.05 (1.82)	2.32 (2.09)	2.26 (1.96)	2.37 (2.16)	2.28 (1.94)	2.22 (1.89)	2.26 (1.96)	2.22 (1.90)	2.29 (1.94)	2.27 (2.01)	2.26 (1.86)
PH												
Mean (SD)	7.32 (0.11)	7.32 (0.12)	7.31 (0.12)	7.31 (0.12)	7.31 (0.12)	7.32 (0.12)	7.32 (0.12)	7.31 (0.12)	7.33 (0.11)	7.32 (0.12)	7.32 (0.12)	7.32 (0.12)
PO2												
Mean (SD)	132.99 (115.77)	131.90 (113.91)	129.24 (114.75)	129.64 (111.48)	135.09 (117.90)	134.53 (119.34)	131.53 (113.59)	129.95 (112.35)	136.35 (114.73)	132.71 (117.21)	130.68 (113.74)	132.32 (117.53)
PCO2												
Mean (SD)	43.23 (13.09)	43.39 (13.07)	42.04 (12.78)	42.88 (13.88)	41.48 (12.71)	41.99 (13.02)	42.47 (12.79)	42.85 (13.85)	41.14 (11.30)	41.99 (12.82)	42.10 (13.38)	42.23 (13.02)
Lactate												
Mean (SD)	3.14 (2.70)	3.14 (2.69)	3.40 (3.02)	3.38 (2.91)	3.39 (3.10)	3.36 (2.88)	3.20 (2.74)	3.39 (2.82)	3.14 (2.63)	3.26 (2.76)	3.29 (2.66)	3.33 (2.83)
Bicarbonate												
Mean (SD)	20.60 (5.40)	20.62 (5.40)	19.81 (5.86)	20.14 (5.44)	20.05 (5.69)	20.34 (5.10)	20.33 (5.16)	20.15 (5.43)	20.42 (4.89)	20.41 (5.07)	20.26 (5.24)	20.36 (5.07)
CVP (tested)												
NO	1,054 (51.52%)	1,037 (50.68%)	345 (58.28%)	332 (56.08%)	277 (58.32%)	269 (56.63%)	331 (55.91%)	332 (56.08%)	270 (57.08%)	268 (56.66%)	254 (56.19%)	254 (56.19%)
YES	992 (48.48%)	1,009 (49.32%)	247 (41.72%)	260 (43.92%)	198 (41.68%)	206 (43.37%)	261 (44.09%)	260 (43.92%)	203 (42.92%)	205 (43.34%)	198 (43.81%)	198 (43.81%)
BNP (tested)												
NO	1918 (93.74%)	1922 (93.94%)	544 (91.89%)	548 (92.57%)	437 (92.00%)	435 (91.58%)	542 (91.55%)	548 (92.57%)	431 (91.12%)	434 (91.75%)	416 (92.04%)	417 (92.26%)
YES	128 (6.26%)	124 (6.06%)	48 (8.11%)	44 (7.43%)	38 (8.00%)	40 (8.42%)	50 (8.45%)	44 (7.43%)	42 (8.88%)	39 (8.25%)	36 (7.96%)	35 (7.74%)
Troponin (tested)												
NO	1,206 (58.94%)	1,188 (58.06%)	312 (52.70%)	318 (53.72%)	248 (52.21%)	251 (52.84%)	326 (55.07%)	319 (53.89%)	260 (54.97%)	251 (53.07%)	235 (51.99%)	242 (53.54%)
YES	840 (41.06%)	858 (41.94%)	280 (47.30%)	274 (46.28%)	227 (47.79%)	224 (47.16%)	266 (44.93%)	273 (46.11%)	213 (45.03%)	222 (46.93%)	217 (48.01%)	210 (46.46%)
Creatinine kinase (tested)												
NO	1,127 (55.08%)	1,121 (54.79%)	301 (50.84%)	301 (50.84%)	247 (52.00%)	249 (52.42%)	304 (51.35%)	302 (51.01%)	257 (54.33%)	249 (52.64%)	228 (50.44%)	236 (52.21%)
YES	919 (44.92%)	925 (45.21%)	291 (49.16%)	291 (49.16%)	228 (48.00%)	226 (47.58%)	288 (48.65%)	290 (48.99%)	216 (45.67%)	224 (47.36%)	224 (49.56%)	216 (47.79%)

### Primary outcome

**Figure 3:**
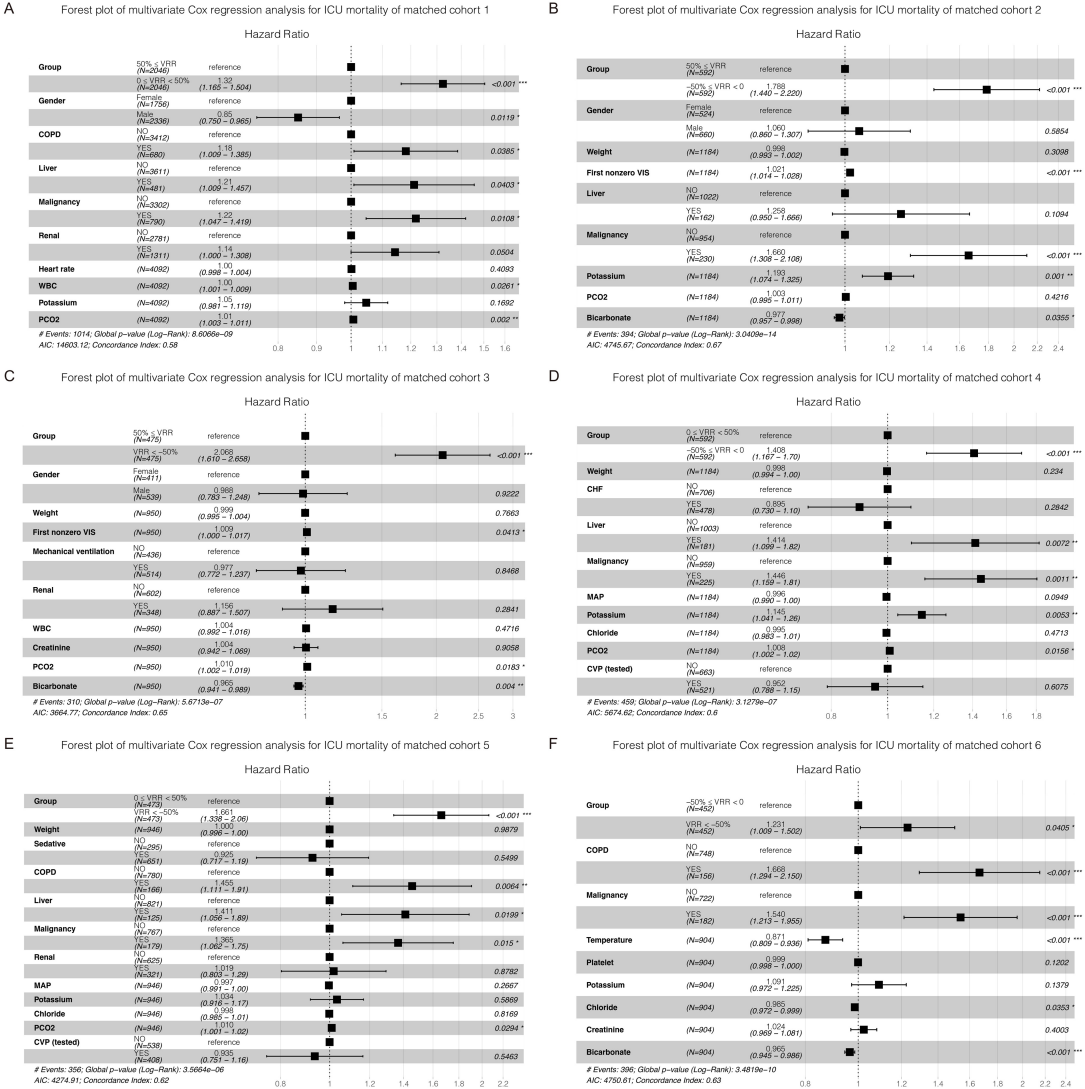
Forest plot of multivariate Cox regression analysis for ICU mortality in each cohort **(A–F)** after pairwise PSM. The differences for each matched cohort were significant.

**Figure 4:**
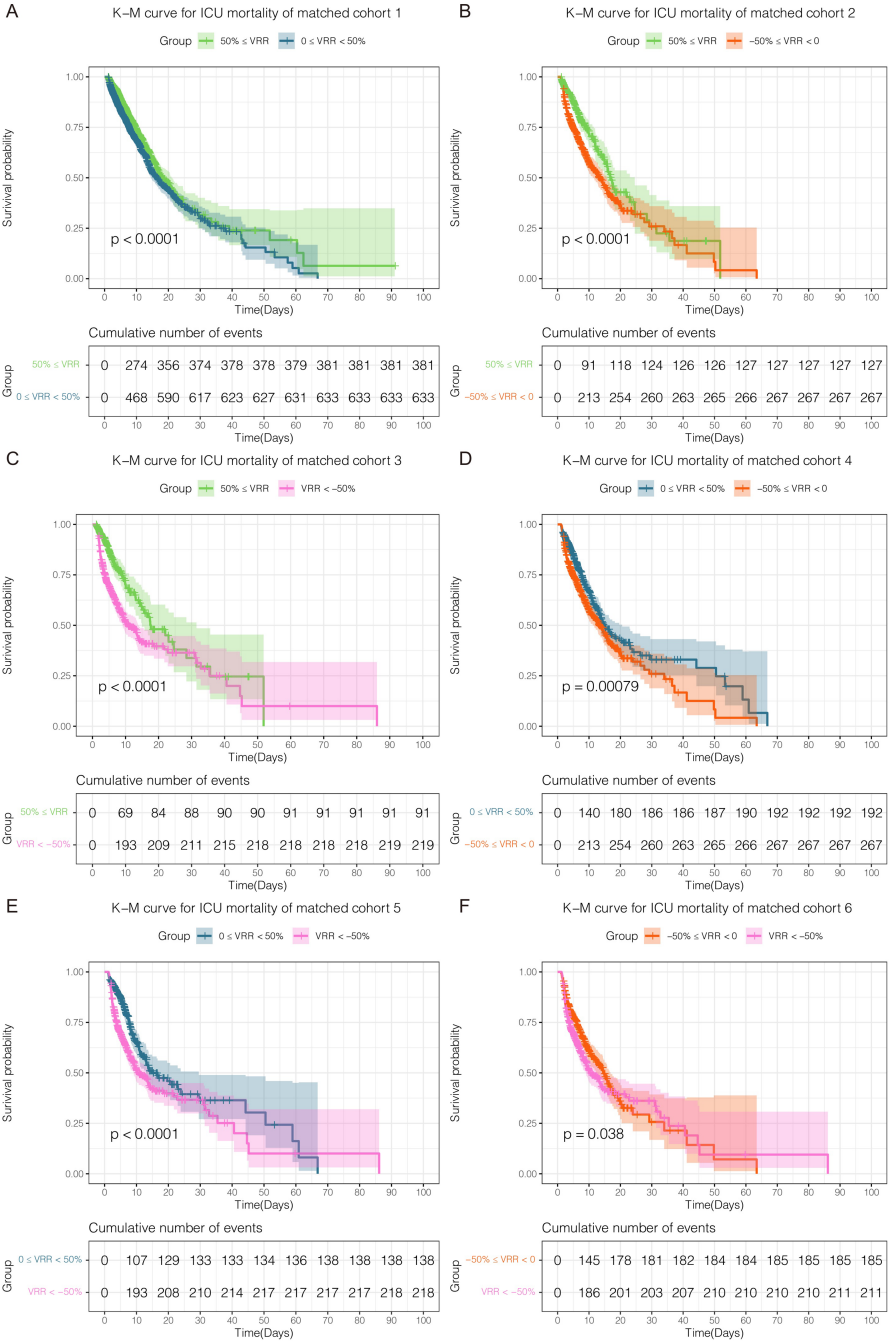
Kaplan–Meier curve for ICU mortality in each cohort **(A–F)** after pairwise PSM. The differences for each matched cohort were significant.

As vital signs, laboratory tests, severity scores, complications, and other covariates were greatly influenced, adjustments were made for these variables in the multivariate Cox regression. The K-M curve and multivariate Cox regression analysis showed a significant beneficial effect in the original cohort with increased VRR in ICU mortality (Supplementary Tables 8, 9; Supplementary Figure 2). In the 6 pairwise matched cohorts after multiple imputation and PSM, multivariate Cox regression analysis revealed patients in group 0 ≤ VRR < 50% [hazard ratio (HR) = 1.32, 95% confidence interval (CI) 1.17–1.50, *p* < 0.001], −50% ≤ VRR < 0 (HR = 1.79, 95% CI 1.44–2.22, p < 0.001), and VRR < −50% (HR = 2.07, 95% CI 1.61–2.66, *p* < 0.001) had a higher risk of mortality compared with 50% ≤ VRR (Figures 3A–C; Supplementary Tables 10, 11); patients in group −50% ≤ VRR < 0 (HR = 1.41, 95% CI 1.17–1.70, *p* < 0.001), and VRR < −50% (HR = 1.66, 95% CI 1.34–2.06, *p* < 0.001) had a higher risk of mortality compared with 0 ≤ VRR < 50% (Figures 3D,E; Supplementary Tables 13, 14); patients in group VRR < −50% (HR = 1.23, 95% CI 1.01–1.50, *p* < 0.05) had a higher risk of mortality compared with −50% ≤ VRR < 0 (Figure 3F; Supplementary Table 15). The K-M estimator of the matched cohorts showed same results (Figures 4A–F). In summary, ICU mortality was increased with decreased VRR values.

### Secondary outcome

**Figure 5:**
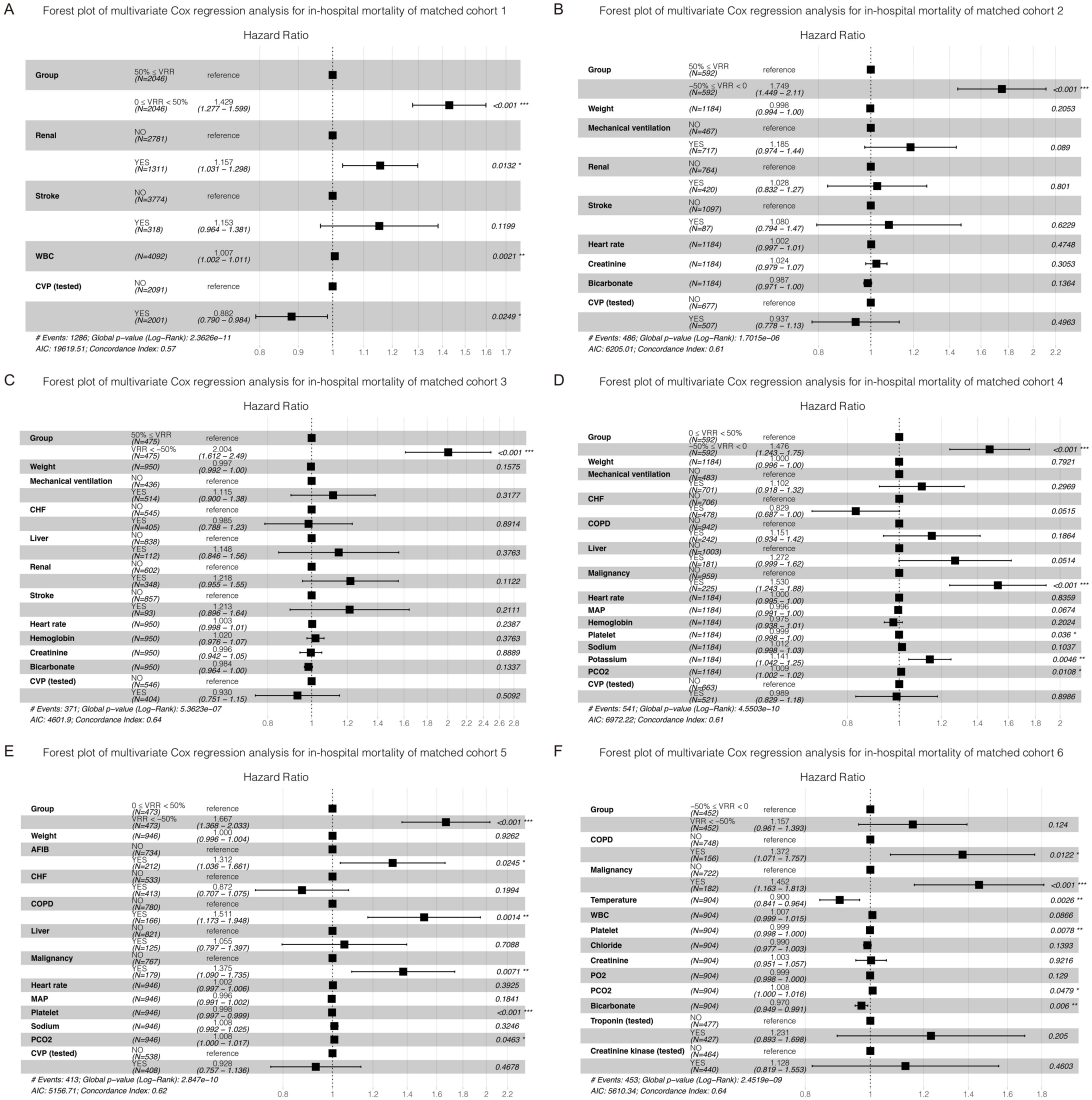
Forest plot of multivariate Cox regression analysis for in-hospital mortality in each cohort after pairwise PSM. Except for matched cohort 6 (**F**, VRR < −50% versus −50% ≤ VRR < 0), there were statistically significant differences between any other groups **(A–E)**.

**Figure 6:**
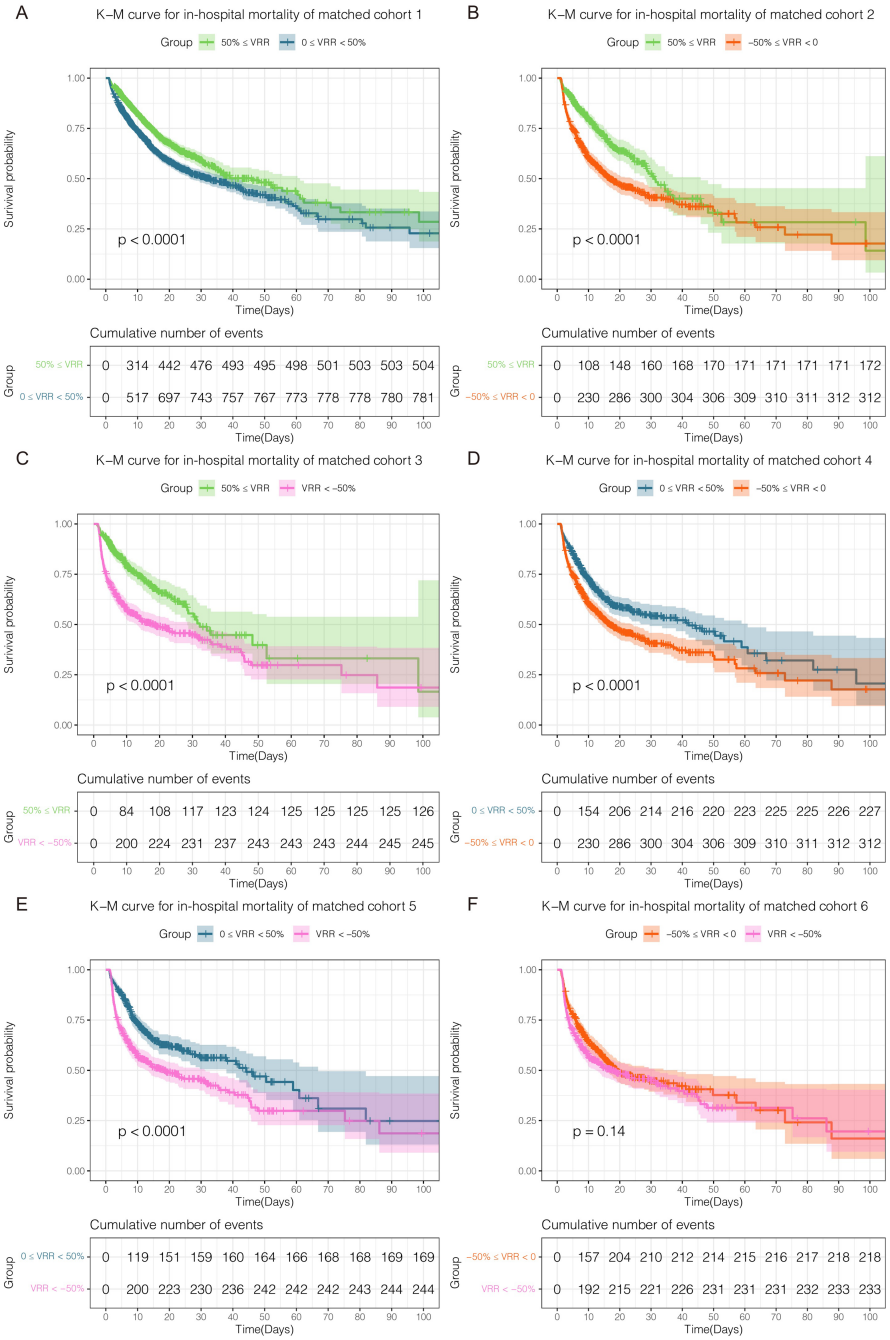
Kaplan–Meier curve for in-hospital mortality in each cohort after pairwise PSM. Except for matched cohort 6 (**F**, VRR < −50% versus −50% ≤ VRR < 0), there were statistically significant differences between any other groups **(A–E)**.

The results for matched cohorts of in-hospital mortality were similar to ICU mortality. Survival analysis indicated patients in group 50% ≤ VRR showed the lowest in-hospital mortality in the original cohort (Supplementary Tables 16, 17; Supplementary Figure 3). For the 6 matched cohorts, multivariate Cox regression analysis revealed significant differences (*p* < 0.001) in pairwise comparisons between any of the groups (Figures 5A–E; Supplementary Tables 18–22), except for VRR < −50% versus −50% ≤ VRR < 0 (Figure 5F; Supplementary Table 23), which had no statistically significant difference for in-hospital mortality (HR 1.16, 95% CI 0.96–1.39, *p* = 0.12). The K-M estimator of the matched cohorts showed similar trends (Figures 6A–F). Although in-hospital mortality was significant non-difference in matched cohort 6, it should be noted that VRR values of both two groups in cohort 6 were negative, implying that once the intensity of hemodynamic support therapy quantified by VIS decreased during 25–48 h compared with 1–24 h (namely VRR > 0), it was inclined to be in lower in-hospital mortality.

### Two doubly robust estimation models for primary and secondary outcomes

To ensure the robustness of the results, as shown in Table 2, two doubly robust estimation models: the survey-weighted generalized linear model and survey-weighted Cox model with all covariates using IPW, were also conducted for ICU and in-hospital mortality in each matched cohort. These findings indicated that an increase in VRR had a significant beneficial effect on survival in ICU or hospital. In terms of ICU mortality and in-hospital mortality, the results of two doubly robust estimation models were similar to those of multivariate Cox regression for in-hospital mortality. Except for matched cohort 6 (VRR < −50% versus −50% ≤ VRR < 0), there were statistically significant differences (*p* < 0.05, Table 2) for ICU mortality and in-hospital mortality between any other groups in the survey-weighted generalized linear model [odds ratio (OR) 1.24, 95% CI 0.94–1.65, *p* = 0.13; OR 1.06, 95% CI 0.80–1.41, *p* = 0.7, respectively. Only for cohort 6] and survey-weighted Cox model [OR 1.16, 95% CI 0.96–1.42, p = 0.13; OR 1.06, 95% CI 0.89–1.28, *p* = 0.5, respectively. Only for cohort 6]. This result may be due to the higher mortality of the two groups in cohort 6. Multivariate Cox regression models for the six matched cohorts mentioned above are also presented in Table 2. All the three models for matched cohort 1 to cohort 5 came to the same conclusion: patients with a higher positive VRR value were highly associated with lower ICU mortality and in-hospital mortality.

**Table 2:** Primary and secondary outcome analysis with three different models: (1) Multivariate Cox regression analysis model, (2) Survey-weighted generalized linear model of with all covariates using IPW; (3) Survey-weighted Cox model with all covariates using IPW.

	Cohort 1	Cohort 2	Cohort 3	Cohort 4	Cohort 5	Cohort 6
ICU mortality						
Multivariate Cox regression [HR (95% CI)]	**1.32 (1.17, 1.50)**	**1.79 (1.44, 2.22)**	**2.07 (1.61, 2.66)**	**1.41 (1.17, 1.70)**	**1.66 (1.34, 2.06)**	**1.23 (1.01, 1.50)**
Survey-weighted generalized linear models [OR (95% CI)]	**2.23 (1.91, 2.60)**	**4.06 (3.09, 5.35)**	**4.58 (3.36, 6.25)**	**1.81 (1.44, 2.27)**	**2.48 (1.91, 3.22)**	1.24 (0.94, 1.65)
Survey-weighted Cox models [OR (95% CI)]	**1.33 (1.17, 1.51)**	**1.82 (1.46, 2.26)**	**1.59 (1.17, 2.16)**	**1.50 (1.28, 1.76)**	**1.68 (1.37, 2.05)**	1.16 (0.96, 1.42)
In-hospital mortality						
Multivariate Cox regression [HR (95% CI)]	**1.43 (1.28, 1.60)**	**1.75 (1.45, 2.11)**	**2.00 (1.61, 2.49)**	**1.48 (1.24, 1.75)**	**1.67 (1.37, 2.03)**	1.16 (0.96, 1.39)
Survey-weighted generalized linear models [OR (95% CI)]	**2.08 (1.80, 2.41)**	**3.77 (2.89, 4.93)**	**3.54 (2.59, 4.83)**	**1.78 (1.42, 2.23)**	**2.14 (1.65, 2.77)**	1.06 (0.80, 1.41)
Survey-weighted Cox models [OR (95% CI)]	**1.50 (1.30, 1.64)**	**2.03 (1.65, 2.51)**	**1.95 (1.53, 2.49)**	**1.45 (1.25, 1.68)**	**1.67 (1.41, 1.99)**	1.06 (0.89, 1.28)

HR, Hazard Ratio; CI, Confidence Interval; OR, Odds Ratio. For each cohort, statistical results of different models in bold have *p* values < 0.05. OR/HR represents risk of mortality in low VRR group relative to high VRR group in each cohort.

## Discussion

In clinical practice, without the state-of-the-art hospital information system (HIS) to collect information and the dose rate of vasoactive agents from infusion pumps, it would be a formidable challenge to obtain real-time VIS data with high accuracy. This means that extensive, quick, and easy assessment of the prognosis of patients with VIS data is a daunting task for ICU medical staff, although compared with dynamic VIS data, we have simplified the richness of data in our XGBoost model with static VIS data obtained within 1–24 h and 25–48 h. Especially in small- and medium-sized ICUs in less developed areas, the phenomenon of information silos between ICU hardware devices and HISs is quite widespread. It should be noted that a sophisticated and well-designed bedside ICU observation chart will help to solve this problem.

Open-source critical care databases such as the MIMIC and eICU databases have made it possible for data science to implement big data in critical care. In recent years, there has been a growing number of studies on prognostic models with supervised learning and deep learning algorithms. In this retrospective, observational evaluation of more than 8,000 patients with septic shock, we first proposed the term VRR to describe the dynamic changes in VIS, which can be used to evaluate the intensity of hemodynamic support. Then, we applied deep learning and machine learning algorithms to process a large amount of dynamic VIS data and fewer static VIS data separately, which objectively and quantitatively proved for the first time that temporal changes in VIS data represented by VRR were highly correlated with ICU mortality of patients with septic shock. To examine VRR as an independent predictor of ICU and in-hospital mortality, we used multivariate Cox regression in different groups of enrolled patients divided by VRR value, and multivariate Cox regression, and two doubly robust estimation models were conducted among subgroups after pairwise PSM. There is no doubt that our extensive data support the empirical conclusion most commonly known by experienced ICU staff that dynamic changes in the dose rates of vasoactive agents in ICU patients with septic shock can be used to assess ICU and in-hospital mortality.

Septic shock is the most severe complication of sepsis and has a high mortality. Vasoactive agents therapy is an essential treatment in hemodynamic support for patients with septic shock, which has become a widespread clinical consensus. Norepinephrine, a powerful α-1 and β-1 adrenergic receptor agonist, can constrict the blood vessels and elevate MAP with minimal impact on heart rate, and it is strongly recommended as the first-line agent over other vasopressors for adults with septic shock. However, excessively high doses of norepinephrine may still increase splanchnic vascular resistance and cause circulatory impairment (30). Dopamine, vasopressin, and epinephrine are typically alternatives or add-on vasoactive agents to norepinephrine in septic shock when MAP levels are inadequate, or whenever the norepinephrine is not available. Dopamine and epinephrine may increase heart rate or induce tachyarrhythmias, while vasopressin may increase the risk of digital ischemia (31). Dobutamine and milrinone were used for cardiac output augmentation by enhancing cardiac contractility and rate in septic shock with myocardial dysfunction to maintain organ perfusion, though it may cause hypotension and tachyarrhythmias. There is increasing discussion regarding the possible benefit of a tailored vasopressor treatment strategy for individual patients (5), and the rational use of vasoactive agents should be emphasized. Collectively, vasoactive agents featuring a narrow therapeutic spectrum could lead to potentially lethal complications. Hence, these drugs need precise therapeutic targets, close monitoring with titration to the minimal efficacious dose, and ought to be weaned as promptly as possible. Additionally, administering vasoactive agents in septic shock requires an individualized approach.

In a multi-center prospective cohort trial involving 1,639 patients, Robert et al. found that an increase in the initial 24 h vasoactive agents dosage of septic shock was associated with an increase in mortality (10). Gaies et al. released the VIS in 2010, which was an updated version of the inotropic score (IS), that included more commonly used vasopressors (adding norepinephrine, vasopressin, and milrinone) compared to the original version (13), thus more comprehensively quantitatively assessing the dosage of vasoactive agents. In clinical practice, multiple vasoactive agents are used in patients with septic shock, and the VIS is undoubtedly a valuable scoring system for hemodynamic management. Vasoactive agents can reveal the severity of septic shock. Many clinical trials have found that early higher VIS was significantly associated with increased mortality among patients with septic shock (14, 32), cardiac surgery (16, 17), and cardiac mechanical circulatory support (33). A single-center retrospective study including 910 adult patients with sepsis suggested that VISmax during the first 6 h of emergency department admission was remarkably associated with 30-day mortality (32). Moreover, VISmax was superior to the cardiovascular component of the SOFA score and initial lactate levels and nearly equivalent to the acute physiology and chronic health evaluation (APACHE) II score (32). Previous studies on the VIS were limited to a single measurement to assess the correlation with mortality. Our research focuses on the effect of changes in the VIS, that is, the reduction in the dose rate of vasoactive agents, on the mortality of patients with septic shock. In this study, using the sophisticated LSTM deep learning algorithm with 1 h-resolution real-time VIS data, we found that temporal changes in the dose rates of vasoactive agents were able to provide clinicians with reliable and up-to-date prognostic information, which may serve as new evidence and be helpful in early and reliable prognostication for septic shock. Through the XGBoost machine learning algorithm, we evaluated fewer VIS data from the static perspective. Overall, we found that the dynamic changes of VIS in the first 48 h after ICU admission, which is a type of point-of-care data and blood-free indicator with no additional financial cost, can be applied to assess patient prognosis.

To the best of our knowledge, this is the first study to quantitatively evaluate the correlation between the reduction intensity of hemodynamic support and mortality among patients with septic shock using VRR, a completely new notion calculated based on the VIS proposed by us. Our study explored high-resolution data for analysis on the large sample size, and confirmed that early vasoactive dose reductions were associated with improved clinical outcomes. In this study, mortality was lowest when VRR was greater than 50%. But there are also some limitations, an important point is that future studies are needed to standardize the protocol further to assess the interaction between fluid volume and reduction of vasoactive agents dosage. In addition, although the VIS is a comprehensive and quantitative evaluation tool for the use of vasoactive agents, the correction factors of these drugs may not strictly conform to the pharmacological effect. Furthermore, patients’ response to different vasopressors is neither uniform nor predictable. Which may impact the outcome remarkably (34). But nevertheless, this is also an effective way to describe the extensive heterogeneity of vasoactive agents therapy. Although all analyses were adjusted for known and possible confounders, it was still possible that there are unknown and residual confounders that our existing models cannot explain.

## Conclusion

In conclusion, VRR is an important indicator affecting the prognosis of the patients among VIS data. Increased VRR value was remarkably associated with lower ICU and in-hospital mortality among patients with septic shock receiving vasoactive–inotropic therapy for more than 24 h. The complexity and severity of the ICU patient’s condition determine the diversity of administration of the vasoactive–inotropic agents. In contrast, the temporal dynamic change of the intensity of hemodynamic support with multiple vasoactive agents has been ignored to some extent due to the past time. They are now quite feasible and free to access, it is absolutely necessary to be evaluated in the clinical practice for optimal clinical care and decision-making.

## Data availability statement

The MIMIC-IV database is publicly available on PhysioNet (https://www.physionet.org/). Concepts codes are available in the MIMIC Code Repository (https://github.com/MIT-LCP/mimic-code/). Codes involved in this study are available on GitHub (https://github.com/ningyile/Septic-Shock-VRR).

## Ethics statement

Y-LN, CS, and W-TC have full access to the MIMIC-IV database. The establishment of this database was approved by the Massachusetts Institute of Technology (Cambridge, MA) and Beth Israel Deaconess Medical Center (Boston, MA), and consent was obtained for the original data collection. Therefore, the ethical approval statement and the need for informed consent were waived for this manuscript.

## Author contributions

CS and W-TC conceived the term VRR and the study, which was designed in detail by Y-LN. The SQL, R, and Python data analysis codes for this study were completed by Y-LN and X-HX. Y-LN led the training of deep learning and machine learning models. Y-LN and CS verified the data and drafted the manuscript. LL, Y-JK, and YM contributed to the data interpretation. X-FL, Z-QY, S-XX, and W-TC were the senior supervisors of this project and revised the manuscript. All authors reviewed and approved the submitted manuscript.

## Funding

This study was supported by the Basic Research Projects Jointly Funding by Municipal Universities (Colleges) of Guangzhou Municipal Science and Technology Bureau (202201020325 and 202102010381), the Rural Science and Technology Mission from the Department of Science and Technology of Guangdong Province (KTPYJ2021027), the National Clinical Research Base of Traditional Chinese Medicine (A6-2-2110100602), the Guangdong Basic and Applied Basic Research Foundation (grant No. 2019A1515011010), the Foundation of Innovative Development Project of the First Affiliated Hospital of Guangzhou University of Chinese Medicine (2017QN05), and the Fund of Guangdong Provincial Traditional Chinese Medicine Bureau Research Project (grant No. 20192018).

## Conflict of interest

Y-LN, CS, and W-TC will apply for a patent for the VRR model developed in this study.

The remaining authors declare that the research was conducted in the absence of any commercial or financial relationships that could be construed as a potential conflict of interest.

## Publisher’s note

All claims expressed in this article are solely those of the authors and do not necessarily represent those of their affiliated organizations, or those of the publisher, the editors and the reviewers. Any product that may be evaluated in this article, or claim that may be made by its manufacturer, is not guaranteed or endorsed by the publisher.

## Abbreviations

VIS: Vasoactive–inotropic score
ICU: intensive care unit
VRR: VIS reduction rate
MIMIC: Medical Information Mart for Intensive Care
SQL: Structured query language
LSTM: Long short-term memory
XGBoost: Extreme gradient boosting
SHAP: SHapley Additive exPlanations
HR: Hazard ratio
OR: Odds ratio
CI: Confidence interval
RNN: Recurrent neural network
PSM: Propensity score matching
K-M: Kaplan–Meier
IPW: Inverse probability weighting
AUROC: Area under the receiver operating characteristic curve
NPV: Negative predictive value
PPV: Positive predictive value
NLR: Negative likelihood ratio
PLR: Positive likelihood ratio
ROC: Receiver operating characteristic
SAPS: Simplified acute physiology score
SOFA: Sequential organ failure assessment
AFIB: Atrial fibrillation
CAD: Coronary artery disease
CHF: Chronic heart failure
COPD: Chronic obstructive pulmonary disease
MAP: mean arterial pressure
WBC: white blood cell
PH: potential of hydrogen
PO2: Partial pressure of oxygen
PCO2: Partial pressure of carbon dioxide
CVP: Central venous pressure
BNP: B-type natriuretic peptide
HIS: Hospital information system
IS: inotropic score
APACHE: Acute physiology and chronic health evaluation
AVP: vasopressin
DA: dopamine
DBA: dobutamine
EPI: epinephrine
MIL: milrinone
NE: norepinephrine
