# Supplementary material for: Tendency of dynamic vasoactive and inotropic medications data as a robust predictor of mortality in patients with septic shock: An analysis of the MIMIC-IV database
Source: Front Cardiovasc Med. 2023 Mar 7;10:1126888. doi: 10.3389/fcvm.2023.1126888 (PMC10112491; doi:10.3389/fcvm.2023.1126888)
Supplement: Supplementary file 1 [file Data_Sheet_1.docx]

**Supplementary Table 1. Basic demographic characteristics of the original cohort**

|  | 50% ≤ VRR (N=5734) | 0 ≤ VRR < 50% (N=2084) | -50% ≤ VRR < 0 (N=593) | VRR < -50% (N=476) | P-value | SMD (Compare to group 50% ≤ VRR, respectively) |
| --- | --- | --- | --- | --- | --- | --- |
| **Age** |  |  |  |  |  |  |
| Mean (SD) | 67.28 (14.75) | 66.90 (15.18) | 66.42 (14.94) | 66.63 (14.62) | 0.4 | [ 0.03, 0.06, 0.04] |
| **Gender** |  |  |  |  |  |  |
| Female | 2393 (41.73%) | 886 (42.51%) | 254 (42.83%) | 200 (42.02%) | 0.9 | [ 0.02, 0.02, 0.01] |
| Male | 3341 (58.27%) | 1198 (57.49%) | 339 (57.17%) | 276 (57.98%) |  |  |
| **Weight** |  |  |  |  |  |  |
| Mean (SD) | 82.50 (24.26) | 83.40 (24.86) | 83.14 (25.21) | 83.28 (26.16) | 0.64 | [ 0.04, 0.03, 0.03] |
| **SAPS II** |  |  |  |  |  |  |
| Mean (SD) | 43.73 (13.84) | 50.03 (15.40) | 51.77 (14.88) | 49.67 (14.82) | <0.05 | [ 0.43, 0.56, 0.42] |
| **SOFA score** |  |  |  |  |  |  |
| Mean (SD) | 9.14 (3.42) | 10.95 (3.79) | 11.66 (3.93) | 11.13 (3.79) | <0.05 | [ 0.5, 0.68, 0.55] |
| **First nonzero VIS** |  |  |  |  |  |  |
| Mean (SD) | 16.60 (53.24) | 15.63 (15.14) | 13.16 (12.23) | 10.72 (11.92) | <0.05 | [ 0.02, 0.09, 0.15] |
| **Mechanical ventilation** |  |  |  |  |  |  |
| NO | 2766 (48.24%) | 834 (40.02%) | 233 (39.29%) | 217 (45.59%) | <0.05 | [ 0.17, 0.18, 0.05] |
| YES | 2968 (51.76%) | 1250 (59.98%) | 360 (60.71%) | 259 (54.41%) |  |  |
| **Sedative** |  |  |  |  |  |  |
| NO | 1894 (33.03%) | 560 (26.87%) | 148 (24.96%) | 146 (30.67%) | <0.05 | [ 0.13, 0.18, 0.05] |
| YES | 3840 (66.97%) | 1524 (73.13%) | 445 (75.04%) | 330 (69.33%) |  |  |
| **AFIB** |  |  |  |  |  |  |
| NO | 4586 (79.98%) | 1676 (80.42%) | 475 (80.10%) | 368 (77.31%) | 0.48 | [ 0.01, 0, 0.07] |
| YES | 1147 (20.00%) | 406 (19.48%) | 118 (19.90%) | 108 (22.69%) |  |  |
| Missing | 1 (0.0%) | 2 (0.1%) | 0 (0%) | 0 (0%) |  |  |
| **CAD** |  |  |  |  |  |  |
| NO | 3614 (63.03%) | 1375 (65.98%) | 380 (64.08%) | 296 (62.18%) | 0.089 | [ 0.06, 0.02, 0.02] |
| YES | 2119 (36.96%) | 707 (33.93%) | 213 (35.92%) | 180 (37.82%) |  |  |
| Missing | 1 (0.0%) | 2 (0.1%) | 0 (0%) | 0 (0%) |  |  |
| **CHF** |  |  |  |  |  |  |
| NO | 3556 (62.02%) | 1218 (58.45%) | 362 (61.05%) | 275 (57.77%) | <0.05 | [ 0.07, 0.02, 0.09] |
| YES | 2177 (37.97%) | 864 (41.46%) | 231 (38.95%) | 201 (42.23%) |  |  |
| Missing | 1 (0.0%) | 2 (0.1%) | 0 (0%) | 0 (0%) |  |  |
| **COPD** |  |  |  |  |  |  |
| NO | 4776 (83.29%) | 1737 (83.35%) | 477 (80.44%) | 394 (82.77%) | 0.34 | [ 0, 0.07, 0.01] |
| YES | 957 (16.69%) | 345 (16.55%) | 116 (19.56%) | 82 (17.23%) |  |  |
| Missing | 1 (0.0%) | 2 (0.1%) | 0 (0%) | 0 (0%) |  |  |
| **Liver** |  |  |  |  |  |  |
| NO | 5291 (92.27%) | 1827 (87.67%) | 510 (86.00%) | 420 (88.24%) | <0.05 | [ 0.15, 0.2, 0.14] |
| YES | 442 (7.71%) | 255 (12.24%) | 83 (14.00%) | 56 (11.76%) |  |  |
| Missing | 1 (0.0%) | 2 (0.1%) | 0 (0%) | 0 (0%) |  |  |
| **Malignancy** |  |  |  |  |  |  |
| NO | 4617 (80.52%) | 1672 (80.23%) | 478 (80.61%) | 380 (79.83%) | 0.98 | [ 0.01, 0, 0.02] |
| YES | 1116 (19.46%) | 410 (19.67%) | 115 (19.39%) | 96 (20.17%) |  |  |
| Missing | 1 (0.0%) | 2 (0.1%) | 0 (0%) | 0 (0%) |  |  |
| **Renal** |  |  |  |  |  |  |
| NO | 4217 (73.54%) | 1428 (68.52%) | 397 (66.95%) | 313 (65.76%) | <0.05 | [ 0.11, 0.14, 0.17] |
| YES | 1516 (26.44%) | 654 (31.38%) | 196 (33.05%) | 163 (34.24%) |  |  |
| Missing | 1 (0.0%) | 2 (0.1%) | 0 (0%) | 0 (0%) |  |  |
| **Stroke** |  |  |  |  |  |  |
| NO | 5334 (93.02%) | 1920 (92.13%) | 547 (92.24%) | 429 (90.13%) | 0.092 | [ 0.03, 0.03, 0.11] |
| YES | 399 (6.96%) | 162 (7.77%) | 46 (7.76%) | 47 (9.87%) |  |  |
| Missing | 1 (0.0%) | 2 (0.1%) | 0 (0%) | 0 (0%) |  |  |
| **Heart rate** |  |  |  |  |  |  |
| Mean (SD) | 91.84 (20.89) | 94.44 (21.69) | 93.65 (20.68) | 93.03 (21.07) | <0.05 | [ 0.12, 0.09, 0.06] |
| **MAP** |  |  |  |  |  |  |
| Mean (SD) | 77.44 (18.58) | 76.26 (19.23) | 75.88 (19.27) | 77.57 (19.30) | <0.05 | [ 0.06, 0.08, 0.01] |
| Missing | 1 (0.0%) | 0 (0%) | 0 (0%) | 0 (0%) |  |  |
| **Temperature** |  |  |  |  |  |  |
| Mean (SD) | 36.70 (1.81) | 36.62 (1.13) | 36.52 (1.31) | 36.55 (1.14) | 0.14 | [ 0.05, 0.11, 0.1] |
| Missing | 558 (9.7%) | 181 (8.7%) | 37 (6.2%) | 47 (9.9%) |  |  |
| **WBC** |  |  |  |  |  |  |
| Mean (SD) | 14.96 (9.63) | 14.90 (10.65) | 14.79 (9.27) | 14.75 (8.92) | 0.88 | [ 0.01, 0.02, 0.02] |
| Missing | 32 (0.6%) | 7 (0.3%) | 1 (0.2%) | 3 (0.6%) |  |  |
| **Hemoglobin** |  |  |  |  |  |  |
| Mean (SD) | 10.06 (2.23) | 10.25 (2.33) | 10.24 (2.23) | 10.26 (2.37) | <0.05 | [ 0.08, 0.08, 0.09] |
| Missing | 29 (0.5%) | 6 (0.3%) | 1 (0.2%) | 2 (0.4%) |  |  |
| **Platelet** |  |  |  |  |  |  |
| Mean (SD) | 200.03 (116.59) | 202.05 (123.42) | 191.28 (120.40) | 188.27 (106.23) | 0.051 | [ 0.02, 0.07, 0.11] |
| Missing | 34 (0.6%) | 6 (0.3%) | 1 (0.2%) | 2 (0.4%) |  |  |
| **Sodium** |  |  |  |  |  |  |
| Mean (SD) | 137.48 (13.13) | 136.90 (6.01) | 137.10 (6.17) | 136.63 (5.82) | <0.05 | [ 0.06, 0.04, 0.08] |
| Missing | 15 (0.3%) | 1 (0.0%) | 1 (0.2%) | 0 (0%) |  |  |
| **Potassium** |  |  |  |  |  |  |
| Mean (SD) | 4.35 (0.90) | 4.33 (0.91) | 4.44 (0.92) | 4.38 (0.90) | <0.05 | [ 0.02, 0.1, 0.03] |
| Missing | 13 (0.2%) | 1 (0.0%) | 1 (0.2%) | 0 (0%) |  |  |
| **Chloride** |  |  |  |  |  |  |
| Mean (SD) | 104.47 (8.06) | 102.97 (7.79) | 102.83 (7.99) | 102.48 (8.07) | <0.05 | [ 0.19, 0.2, 0.25] |
| Missing | 15 (0.3%) | 1 (0.0%) | 1 (0.2%) | 0 (0%) |  |  |
| **Bun** |  |  |  |  |  |  |
| Mean (SD) | 31.38 (23.57) | 37.45 (26.88) | 40.28 (28.68) | 40.23 (29.48) | <0.05 | [ 0.24, 0.34, 0.33] |
| Missing | 17 (0.3%) | 2 (0.1%) | 1 (0.2%) | 0 (0%) |  |  |
| **Creatinine** |  |  |  |  |  |  |
| Mean (SD) | 1.74 (1.71) | 2.07 (1.83) | 2.27 (1.96) | 2.29 (1.94) | <0.05 | [ 0.19, 0.29, 0.3] |
| Missing | 16 (0.3%) | 2 (0.1%) | 2 (0.3%) | 0 (0%) |  |  |
| **PH** |  |  |  |  |  |  |
| Mean (SD) | 7.34 (0.11) | 7.31 (0.12) | 7.31 (0.12) | 7.31 (0.12) | <0.05 | [ 0.21, 0.25, 0.18] |
| Missing | 671 (11.7%) | 149 (7.1%) | 39 (6.6%) | 32 (6.7%) |  |  |
| **PO2** |  |  |  |  |  |  |
| Mean (SD) | 159.48 (131.97) | 129.82 (112.46) | 131.42 (112.95) | 133.60 (117.92) | <0.05 | [ 0.24, 0.23, 0.21] |
| Missing | 762 (13.3%) | 178 (8.5%) | 47 (7.9%) | 36 (7.6%) |  |  |
| **PCO2** |  |  |  |  |  |  |
| Mean (SD) | 43.03 (12.71) | 43.33 (13.00) | 42.57 (13.56) | 41.92 (13.00) | 0.12 | [ 0.02, 0.03, 0.09] |
| Missing | 763 (13.3%) | 179 (8.6%) | 47 (7.9%) | 36 (7.6%) |  |  |
| **Lactate** |  |  |  |  |  |  |
| Mean (SD) | 2.75 (2.23) | 3.25 (2.81) | 3.47 (3.00) | 3.38 (2.89) | <0.05 | [ 0.2, 0.27, 0.24] |
| Missing | 502 (8.8%) | 121 (5.8%) | 38 (6.4%) | 28 (5.9%) |  |  |
| **Bicarbonate** |  |  |  |  |  |  |
| Mean (SD) | 21.26 (4.85) | 20.55 (5.42) | 20.14 (5.44) | 20.36 (5.11) | <0.05 | [ 0.14, 0.22, 0.18] |
| Missing | 15 (0.3%) | 1 (0.0%) | 1 (0.2%) | 0 (0%) |  |  |
| **CVP (tested)** |  |  |  |  |  |  |
| NO | 2869 (50.03%) | 1058 (50.77%) | 333 (56.16%) | 270 (56.72%) | <0.05 | [ 0.01, 0.12, 0.13] |
| YES | 2865 (49.97%) | 1026 (49.23%) | 260 (43.84%) | 206 (43.28%) |  |  |
| **BNP (tested)** |  |  |  |  |  |  |
| NO | 5418 (94.49%) | 1955 (93.81%) | 549 (92.58%) | 436 (91.60%) | <0.05 | [ 0.03, 0.08, 0.11] |
| YES | 316 (5.51%) | 129 (6.19%) | 44 (7.42%) | 40 (8.40%) |  |  |
| **Troponin (tested)** |  |  |  |  |  |  |
| NO | 3743 (65.28%) | 1210 (58.06%) | 319 (53.79%) | 252 (52.94%) | <0.05 | [ 0.15, 0.24, 0.25] |
| YES | 1991 (34.72%) | 874 (41.94%) | 274 (46.21%) | 224 (47.06%) |  |  |
| **Creatinine kinase (tested)** |  |  |  |  |  |  |
| NO | 3608 (62.92%) | 1140 (54.70%) | 302 (50.93%) | 250 (52.52%) | <0.05 | [ 0.17, 0.24, 0.21] |
| YES | 2126 (37.08%) | 944 (45.30%) | 291 (49.07%) | 226 (47.48%) |  |  |

**Supplementary Table 2. Baseline Characteristics Before and After Propensity Score Matching of Cohort 1**

|  | Before matching | | | |  | After matching | | | |
| --- | --- | --- | --- | --- | --- | --- | --- | --- | --- |
|  | 50% ≤ VRR (N=5734) | 0 ≤ VRR < 50% (N=2084) | P-value | SMD |  | 50% ≤ VRR (N=2046) | 0 ≤ VRR < 50% (N=2046) | P-value | SMD |
| **Age** |  |  |  |  |  |  |  |  |  |
| Mean (SD) | 67.28 (14.75) | 66.90 (15.18) | 0.37 | 0.03 |  | 67.10 (15.06) | 67.01 (15.17) | 0.78 | 0.01 |
| **Gender** |  |  |  |  |  |  |  |  |  |
| Female | 2393 (41.73%) | 886 (42.51%) | 0.55 | 0.02 |  | 888 (43.40%) | 868 (42.42%) | 0.55 | 0.02 |
| Male | 3341 (58.27%) | 1198 (57.49%) |  |  |  | 1158 (56.60%) | 1178 (57.58%) |  |  |
| **Weight** |  |  |  |  |  |  |  |  |  |
| Mean (SD) | 82.50 (24.26) | 83.40 (24.86) | 0.22 | 0.04 |  | 83.34 (24.17) | 83.30 (24.87) | 0.81 | 0 |
| **SAPS II** |  |  |  |  |  |  |  |  |  |
| Mean (SD) | 43.73 (13.84) | 50.03 (15.40) | <0.05 | 0.43 |  | 49.40 (14.68) | 49.58 (15.01) | 0.78 | 0.01 |
| **SOFA score** |  |  |  |  |  |  |  |  |  |
| Mean (SD) | 9.14 (3.42) | 10.95 (3.79) | <0.05 | 0.5 |  | 10.78 (3.64) | 10.82 (3.67) | 0.37 | 0.01 |
| **First nonzero VIS** |  |  |  |  |  |  |  |  |  |
| Mean (SD) | 16.60 (53.24) | 15.63 (15.14) | <0.05 | 0.02 |  | 15.80 (17.21) | 15.61 (15.15) | 0.27 | 0.01 |
| **Mechanical ventilation** |  |  |  |  |  |  |  |  |  |
| NO | 2766 (48.24%) | 834 (40.02%) | <0.05 | 0.17 |  | 828 (40.47%) | 827 (40.42%) | 1 | 0 |
| YES | 2968 (51.76%) | 1250 (59.98%) |  |  |  | 1218 (59.53%) | 1219 (59.58%) |  |  |
| **Sedative** |  |  |  |  |  |  |  |  |  |
| NO | 1894 (33.03%) | 560 (26.87%) | <0.05 | 0.13 |  | 549 (26.83%) | 558 (27.27%) | 0.78 | 0.01 |
| YES | 3840 (66.97%) | 1524 (73.13%) |  |  |  | 1497 (73.17%) | 1488 (72.73%) |  |  |
| **AFIB** |  |  |  |  |  |  |  |  |  |
| NO | 4587 (80.00%) | 1678 (80.52%) | 0.63 | 0.01 |  | 1641 (80.21%) | 1644 (80.35%) | 0.94 | 0 |
| YES | 1147 (20.00%) | 406 (19.48%) |  |  |  | 405 (19.79%) | 402 (19.65%) |  |  |
| **CAD** |  |  |  |  |  |  |  |  |  |
| NO | 3615 (63.04%) | 1376 (66.03%) | <0.05 | 0.06 |  | 1346 (65.79%) | 1340 (65.49%) | 0.87 | 0.01 |
| YES | 2119 (36.96%) | 708 (33.97%) |  |  |  | 700 (34.21%) | 706 (34.51%) |  |  |
| **CHF** |  |  |  |  |  |  |  |  |  |
| NO | 3557 (62.03%) | 1219 (58.49%) | <0.05 | 0.07 |  | 1169 (57.14%) | 1190 (58.16%) | 0.53 | 0.02 |
| YES | 2177 (37.97%) | 865 (41.51%) |  |  |  | 877 (42.86%) | 856 (41.84%) |  |  |
| **COPD** |  |  |  |  |  |  |  |  |  |
| NO | 4777 (83.31%) | 1739 (83.45%) | 0.91 | 0 |  | 1707 (83.43%) | 1705 (83.33%) | 0.97 | 0 |
| YES | 957 (16.69%) | 345 (16.55%) |  |  |  | 339 (16.57%) | 341 (16.67%) |  |  |
| **Liver** |  |  |  |  |  |  |  |  |  |
| NO | 5292 (92.29%) | 1829 (87.76%) | <0.05 | 0.15 |  | 1804 (88.17%) | 1807 (88.32%) | 0.92 | 0 |
| YES | 442 (7.71%) | 255 (12.24%) |  |  |  | 242 (11.83%) | 239 (11.68%) |  |  |
| **Malignancy** |  |  |  |  |  |  |  |  |  |
| NO | 4618 (80.54%) | 1673 (80.28%) | 0.82 | 0.01 |  | 1655 (80.89%) | 1647 (80.50%) | 0.78 | 0.01 |
| YES | 1116 (19.46%) | 411 (19.72%) |  |  |  | 391 (19.11%) | 399 (19.50%) |  |  |
| **Renal** |  |  |  |  |  |  |  |  |  |
| NO | 4217 (73.54%) | 1430 (68.62%) | <0.05 | 0.11 |  | 1379 (67.40%) | 1402 (68.52%) | 0.46 | 0.02 |
| YES | 1517 (26.46%) | 654 (31.38%) |  |  |  | 667 (32.60%) | 644 (31.48%) |  |  |
| **Stroke** |  |  |  |  |  |  |  |  |  |
| NO | 5335 (93.04%) | 1922 (92.23%) | 0.24 | 0.03 |  | 1889 (92.33%) | 1885 (92.13%) | 0.86 | 0.01 |
| YES | 399 (6.96%) | 162 (7.77%) |  |  |  | 157 (7.67%) | 161 (7.87%) |  |  |
| **Heart rate** |  |  |  |  |  |  |  |  |  |
| Mean (SD) | 91.84 (20.89) | 94.44 (21.69) | <0.05 | 0.12 |  | 94.11 (22.25) | 94.18 (21.58) | 0.52 | 0 |
| **MAP** |  |  |  |  |  |  |  |  |  |
| Mean (SD) | 77.44 (18.58) | 76.26 (19.23) | <0.05 | 0.06 |  | 76.56 (18.73) | 76.46 (19.27) | 0.45 | 0.01 |
| **Temperature** |  |  |  |  |  |  |  |  |  |
| Mean (SD) | 36.66 (1.76) | 36.60 (1.13) | 0.6 | 0.04 |  | 36.58 (1.10) | 36.61 (1.13) | 0.43 | 0.02 |
| **WBC** |  |  |  |  |  |  |  |  |  |
| Mean (SD) | 14.97 (9.64) | 14.91 (10.64) | 0.53 | 0.01 |  | 15.14 (9.92) | 14.95 (10.68) | 0.53 | 0.02 |
| **Hemoglobin** |  |  |  |  |  |  |  |  |  |
| Mean (SD) | 10.06 (2.23) | 10.25 (2.33) | <0.05 | 0.08 |  | 10.28 (2.40) | 10.26 (2.32) | 0.55 | 0.01 |
| **Platelet** |  |  |  |  |  |  |  |  |  |
| Mean (SD) | 200.05 (116.52) | 202.32 (123.63) | 0.75 | 0.02 |  | 201.98 (123.26) | 203.76 (123.74) | 0.43 | 0.01 |
| **Sodium** |  |  |  |  |  |  |  |  |  |
| Mean (SD) | 137.48 (13.12) | 136.90 (6.01) | <0.05 | 0.06 |  | 137.01 (6.12) | 136.96 (5.93) | 0.67 | 0.01 |
| **Potassium** |  |  |  |  |  |  |  |  |  |
| Mean (SD) | 4.35 (0.90) | 4.33 (0.91) | 0.24 | 0.02 |  | 4.33 (0.91) | 4.32 (0.91) | 0.49 | 0.01 |
| **Chloride** |  |  |  |  |  |  |  |  |  |
| Mean (SD) | 104.47 (8.06) | 102.96 (7.80) | <0.05 | 0.19 |  | 103.22 (7.64) | 103.08 (7.72) | 0.45 | 0.02 |
| **Bun** |  |  |  |  |  |  |  |  |  |
| Mean (SD) | 31.37 (23.56) | 37.46 (26.88) | <0.05 | 0.24 |  | 37.42 (27.32) | 37.12 (26.53) | 0.79 | 0.01 |
| **Creatinine** |  |  |  |  |  |  |  |  |  |
| Mean (SD) | 1.74 (1.71) | 2.07 (1.83) | <0.05 | 0.19 |  | 2.07 (1.84) | 2.05 (1.82) | 0.94 | 0.01 |
| **PH** |  |  |  |  |  |  |  |  |  |
| Mean (SD) | 7.34 (0.11) | 7.32 (0.12) | <0.05 | 0.2 |  | 7.32 (0.11) | 7.32 (0.12) | 0.59 | 0.01 |
| **PO2** |  |  |  |  |  |  |  |  |  |
| Mean (SD) | 156.31 (130.00) | 130.96 (113.28) | <0.05 | 0.21 |  | 132.99 (115.77) | 131.90 (113.91) | 0.75 | 0.01 |
| **PCO2** |  |  |  |  |  |  |  |  |  |
| Mean (SD) | 42.96 (12.65) | 43.31 (13.07) | 0.65 | 0.03 |  | 43.23 (13.09) | 43.39 (13.07) | 0.87 | 0.01 |
| **Lactate** |  |  |  |  |  |  |  |  |  |
| Mean (SD) | 2.72 (2.19) | 3.21 (2.77) | <0.05 | 0.2 |  | 3.14 (2.70) | 3.14 (2.69) | 0.84 | 0 |
| **Bicarbonate** |  |  |  |  |  |  |  |  |  |
| Mean (SD) | 21.26 (4.84) | 20.55 (5.42) | <0.05 | 0.14 |  | 20.60 (5.40) | 20.62 (5.40) | 0.76 | 0 |
| **CVP (tested)** |  |  |  |  |  |  |  |  |  |
| NO | 2869 (50.03%) | 1058 (50.77%) | 0.58 | 0.01 |  | 1054 (51.52%) | 1037 (50.68%) | 0.62 | 0.02 |
| YES | 2865 (49.97%) | 1026 (49.23%) |  |  |  | 992 (48.48%) | 1009 (49.32%) |  |  |
| **BNP (tested)** |  |  |  |  |  |  |  |  |  |
| NO | 5418 (94.49%) | 1955 (93.81%) | 0.28 | 0.03 |  | 1918 (93.74%) | 1922 (93.94%) | 0.85 | 0.01 |
| YES | 316 (5.51%) | 129 (6.19%) |  |  |  | 128 (6.26%) | 124 (6.06%) |  |  |
| **Troponin (tested)** |  |  |  |  |  |  |  |  |  |
| NO | 3743 (65.28%) | 1210 (58.06%) | <0.05 | 0.15 |  | 1206 (58.94%) | 1188 (58.06%) | 0.59 | 0.02 |
| YES | 1991 (34.72%) | 874 (41.94%) |  |  |  | 840 (41.06%) | 858 (41.94%) |  |  |
| **Creatinine kinase (tested)** |  |  |  |  |  |  |  |  |  |
| NO | 3608 (62.92%) | 1140 (54.70%) | <0.05 | 0.17 |  | 1127 (55.08%) | 1121 (54.79%) | 0.88 | 0.01 |
| YES | 2126 (37.08%) | 944 (45.30%) |  |  |  | 919 (44.92%) | 925 (45.21%) |  |  |

**Supplementary Table 3. Baseline Characteristics Before and After Propensity Score Matching of Cohort 2**

|  | Before matching | | | |  | After matching | | | |
| --- | --- | --- | --- | --- | --- | --- | --- | --- | --- |
|  | 50% ≤ VRR (N=5734) | -50% ≤ VRR < 0 (N=593) | P-value | SMD |  | 50% ≤ VRR (N=592) | -50% ≤ VRR < 0 (N=592) | P-value | SMD |
| **Age** |  |  |  |  |  |  |  |  |  |
| Mean (SD) | 67.28 (14.75) | 66.42 (14.94) | 0.16 | 0.06 |  | 66.99 (14.78) | 66.43 (14.95) | 0.53 | 0.04 |
| **Gender** |  |  |  |  |  |  |  |  |  |
| Female | 2393 (41.73%) | 254 (42.83%) | 0.64 | 0.02 |  | 270 (45.61%) | 254 (42.91%) | 0.38 | 0.05 |
| Male | 3341 (58.27%) | 339 (57.17%) |  |  |  | 322 (54.39%) | 338 (57.09%) |  |  |
| **Weight** |  |  |  |  |  |  |  |  |  |
| Mean (SD) | 82.50 (24.26) | 83.14 (25.21) | 0.91 | 0.03 |  | 82.92 (24.80) | 83.09 (25.21) | 0.96 | 0.01 |
| **SAPS II** |  |  |  |  |  |  |  |  |  |
| Mean (SD) | 43.73 (13.84) | 51.77 (14.88) | <0.05 | 0.56 |  | 51.37 (15.85) | 51.71 (14.82) | 0.41 | 0.02 |
| **SOFA score** |  |  |  |  |  |  |  |  |  |
| Mean (SD) | 9.14 (3.42) | 11.66 (3.93) | <0.05 | 0.68 |  | 11.48 (3.70) | 11.64 (3.91) | 0.38 | 0.04 |
| **First nonzero VIS** |  |  |  |  |  |  |  |  |  |
| Mean (SD) | 16.60 (53.24) | 13.16 (12.23) | 0.77 | 0.09 |  | 13.59 (13.11) | 13.18 (12.23) | 0.85 | 0.03 |
| **Mechanical ventilation** |  |  |  |  |  |  |  |  |  |
| NO | 2766 (48.24%) | 233 (39.29%) | <0.05 | 0.18 |  | 235 (39.70%) | 232 (39.19%) | 0.91 | 0.01 |
| YES | 2968 (51.76%) | 360 (60.71%) |  |  |  | 357 (60.30%) | 360 (60.81%) |  |  |
| **Sedative** |  |  |  |  |  |  |  |  |  |
| NO | 1894 (33.03%) | 148 (24.96%) | <0.05 | 0.18 |  | 139 (23.48%) | 148 (25.00%) | 0.59 | 0.04 |
| YES | 3840 (66.97%) | 445 (75.04%) |  |  |  | 453 (76.52%) | 444 (75.00%) |  |  |
| **AFIB** |  |  |  |  |  |  |  |  |  |
| NO | 4587 (80.00%) | 475 (80.10%) | 0.99 | 0 |  | 477 (80.57%) | 474 (80.07%) | 0.88 | 0.01 |
| YES | 1147 (20.00%) | 118 (19.90%) |  |  |  | 115 (19.43%) | 118 (19.93%) |  |  |
| **CAD** |  |  |  |  |  |  |  |  |  |
| NO | 3615 (63.04%) | 380 (64.08%) | 0.65 | 0.02 |  | 371 (62.67%) | 379 (64.02%) | 0.67 | 0.03 |
| YES | 2119 (36.96%) | 213 (35.92%) |  |  |  | 221 (37.33%) | 213 (35.98%) |  |  |
| **CHF** |  |  |  |  |  |  |  |  |  |
| NO | 3556 (62.02%) | 362 (61.05%) | 0.68 | 0.02 |  | 351 (59.29%) | 361 (60.98%) | 0.59 | 0.03 |
| YES | 2178 (37.98%) | 231 (38.95%) |  |  |  | 241 (40.71%) | 231 (39.02%) |  |  |
| **COPD** |  |  |  |  |  |  |  |  |  |
| NO | 4776 (83.29%) | 477 (80.44%) | 0.088 | 0.07 |  | 485 (81.93%) | 476 (80.41%) | 0.55 | 0.04 |
| YES | 958 (16.71%) | 116 (19.56%) |  |  |  | 107 (18.07%) | 116 (19.59%) |  |  |
| **Liver** |  |  |  |  |  |  |  |  |  |
| NO | 5291 (92.27%) | 510 (86.00%) | <0.05 | 0.2 |  | 513 (86.66%) | 509 (85.98%) | 0.8 | 0.02 |
| YES | 443 (7.73%) | 83 (14.00%) |  |  |  | 79 (13.34%) | 83 (14.02%) |  |  |
| **Malignancy** |  |  |  |  |  |  |  |  |  |
| NO | 4617 (80.52%) | 478 (80.61%) | 1 | 0 |  | 477 (80.57%) | 477 (80.57%) | 1 | 0 |
| YES | 1117 (19.48%) | 115 (19.39%) |  |  |  | 115 (19.43%) | 115 (19.43%) |  |  |
| **Renal** |  |  |  |  |  |  |  |  |  |
| NO | 4217 (73.54%) | 397 (66.95%) | <0.05 | 0.14 |  | 368 (62.16%) | 396 (66.89%) | 0.1 | 0.1 |
| YES | 1517 (26.46%) | 196 (33.05%) |  |  |  | 224 (37.84%) | 196 (33.11%) |  |  |
| **Stroke** |  |  |  |  |  |  |  |  |  |
| NO | 5335 (93.04%) | 547 (92.24%) | 0.52 | 0.03 |  | 551 (93.07%) | 546 (92.23%) | 0.66 | 0.03 |
| YES | 399 (6.96%) | 46 (7.76%) |  |  |  | 41 (6.93%) | 46 (7.77%) |  |  |
| **Heart rate** |  |  |  |  |  |  |  |  |  |
| Mean (SD) | 91.84 (20.89) | 93.65 (20.68) | <0.05 | 0.09 |  | 93.15 (22.16) | 93.64 (20.70) | 0.31 | 0.02 |
| **MAP** |  |  |  |  |  |  |  |  |  |
| Mean (SD) | 77.44 (18.58) | 75.88 (19.27) | <0.05 | 0.08 |  | 76.51 (18.61) | 75.77 (19.10) | 0.41 | 0.04 |
| **Temperature** |  |  |  |  |  |  |  |  |  |
| Mean (SD) | 36.67 (1.76) | 36.52 (1.29) | 0.3 | 0.1 |  | 36.45 (1.21) | 36.52 (1.29) | 0.078 | 0.06 |
| **WBC** |  |  |  |  |  |  |  |  |  |
| Mean (SD) | 14.97 (9.65) | 14.77 (9.27) | 0.65 | 0.02 |  | 14.98 (9.31) | 14.77 (9.28) | 0.86 | 0.02 |
| **Hemoglobin** |  |  |  |  |  |  |  |  |  |
| Mean (SD) | 10.06 (2.23) | 10.24 (2.23) | 0.083 | 0.08 |  | 10.16 (2.40) | 10.25 (2.23) | 0.47 | 0.04 |
| **Platelet** |  |  |  |  |  |  |  |  |  |
| Mean (SD) | 199.96 (116.45) | 191.18 (120.33) | <0.05 | 0.07 |  | 192.17 (127.70) | 191.41 (120.29) | 0.76 | 0.01 |
| **Sodium** |  |  |  |  |  |  |  |  |  |
| Mean (SD) | 137.48 (13.12) | 137.10 (6.17) | 0.74 | 0.04 |  | 136.90 (6.28) | 137.09 (6.17) | 0.31 | 0.03 |
| **Potassium** |  |  |  |  |  |  |  |  |  |
| Mean (SD) | 4.35 (0.90) | 4.44 (0.92) | <0.05 | 0.1 |  | 4.42 (0.94) | 4.44 (0.92) | 0.66 | 0.02 |
| **Chloride** |  |  |  |  |  |  |  |  |  |
| Mean (SD) | 104.48 (8.06) | 102.84 (7.98) | <0.05 | 0.2 |  | 103.09 (8.09) | 102.85 (7.99) | 0.46 | 0.03 |
| **Bun** |  |  |  |  |  |  |  |  |  |
| Mean (SD) | 31.37 (23.55) | 40.23 (28.67) | <0.05 | 0.34 |  | 41.72 (31.10) | 40.21 (28.69) | 0.55 | 0.05 |
| **Creatinine** |  |  |  |  |  |  |  |  |  |
| Mean (SD) | 1.74 (1.71) | 2.26 (1.95) | <0.05 | 0.28 |  | 2.32 (2.09) | 2.26 (1.96) | 0.84 | 0.03 |
| **PH** |  |  |  |  |  |  |  |  |  |
| Mean (SD) | 7.34 (0.11) | 7.31 (0.12) | <0.05 | 0.25 |  | 7.31 (0.12) | 7.31 (0.12) | 0.79 | 0.03 |
| **PO2** |  |  |  |  |  |  |  |  |  |
| Mean (SD) | 156.89 (130.46) | 129.52 (111.42) | <0.05 | 0.23 |  | 129.24 (114.75) | 129.64 (111.48) | 0.47 | 0 |
| **PCO2** |  |  |  |  |  |  |  |  |  |
| Mean (SD) | 42.93 (12.64) | 42.87 (13.86) | 0.31 | 0 |  | 42.04 (12.78) | 42.88 (13.88) | 0.82 | 0.06 |
| **Lactate** |  |  |  |  |  |  |  |  |  |
| Mean (SD) | 2.72 (2.18) | 3.40 (2.93) | <0.05 | 0.26 |  | 3.40 (3.02) | 3.38 (2.91) | 0.5 | 0.01 |
| **Bicarbonate** |  |  |  |  |  |  |  |  |  |
| Mean (SD) | 21.25 (4.85) | 20.14 (5.44) | <0.05 | 0.22 |  | 19.81 (5.86) | 20.14 (5.44) | 0.55 | 0.06 |
| **CVP (tested)** |  |  |  |  |  |  |  |  |  |
| NO | 2869 (50.03%) | 333 (56.16%) | <0.05 | 0.12 |  | 345 (58.28%) | 332 (56.08%) | 0.48 | 0.04 |
| YES | 2865 (49.97%) | 260 (43.84%) |  |  |  | 247 (41.72%) | 260 (43.92%) |  |  |
| **BNP (tested)** |  |  |  |  |  |  |  |  |  |
| NO | 5418 (94.49%) | 549 (92.58%) | 0.069 | 0.08 |  | 544 (91.89%) | 548 (92.57%) | 0.74 | 0.03 |
| YES | 316 (5.51%) | 44 (7.42%) |  |  |  | 48 (8.11%) | 44 (7.43%) |  |  |
| **Troponin (tested)** |  |  |  |  |  |  |  |  |  |
| NO | 3743 (65.28%) | 319 (53.79%) | <0.05 | 0.24 |  | 312 (52.70%) | 318 (53.72%) | 0.77 | 0.02 |
| YES | 1991 (34.72%) | 274 (46.21%) |  |  |  | 280 (47.30%) | 274 (46.28%) |  |  |
| **Creatinine kinase (tested)** |  |  |  |  |  |  |  |  |  |
| NO | 3608 (62.92%) | 302 (50.93%) | <0.05 | 0.24 |  | 301 (50.84%) | 301 (50.84%) | 1 | 0 |
| YES | 2126 (37.08%) | 291 (49.07%) |  |  |  | 291 (49.16%) | 291 (49.16%) |  |  |

**Supplementary Table 4. Baseline Characteristics Before and After Propensity Score Matching of Cohort 3**

|  | Before matching | | | |  | After matching | | | |
| --- | --- | --- | --- | --- | --- | --- | --- | --- | --- |
|  | 50% ≤ VRR (N=5734) | VRR < -50% (N=476) | P-value | SMD |  | 50% ≤ VRR (N=475) | VRR < -50% (N=475) | P-value | SMD |
| **Age** |  |  |  |  |  |  |  |  |  |
| Mean (SD) | 67.28 (14.75) | 66.63 (14.62) | 0.36 | 0.04 |  | 66.91 (14.51) | 66.63 (14.63) | 0.81 | 0.02 |
| **Gender** |  |  |  |  |  |  |  |  |  |
| Female | 2393 (41.73%) | 200 (42.02%) | 0.94 | 0.01 |  | 212 (44.63%) | 199 (41.89%) | 0.43 | 0.06 |
| Male | 3341 (58.27%) | 276 (57.98%) |  |  |  | 263 (55.37%) | 276 (58.11%) |  |  |
| **Weight** |  |  |  |  |  |  |  |  |  |
| Mean (SD) | 82.50 (24.26) | 83.28 (26.16) | 0.6 | 0.03 |  | 83.28 (24.60) | 83.25 (26.18) | 0.93 | 0 |
| **SAPS II** |  |  |  |  |  |  |  |  |  |
| Mean (SD) | 43.73 (13.84) | 49.67 (14.82) | <0.05 | 0.42 |  | 49.29 (15.59) | 49.56 (14.62) | 0.52 | 0.02 |
| **SOFA score** |  |  |  |  |  |  |  |  |  |
| Mean (SD) | 9.14 (3.42) | 11.13 (3.79) | <0.05 | 0.55 |  | 11.05 (3.82) | 11.11 (3.76) | 0.65 | 0.02 |
| **First nonzero VIS** |  |  |  |  |  |  |  |  |  |
| Mean (SD) | 16.60 (53.24) | 10.72 (11.92) | <0.05 | 0.15 |  | 11.67 (12.67) | 10.73 (11.93) | 0.24 | 0.08 |
| **Mechanical ventilation** |  |  |  |  |  |  |  |  |  |
| NO | 2766 (48.24%) | 217 (45.59%) | 0.29 | 0.05 |  | 219 (46.11%) | 217 (45.68%) | 0.95 | 0.01 |
| YES | 2968 (51.76%) | 259 (54.41%) |  |  |  | 256 (53.89%) | 258 (54.32%) |  |  |
| **Sedative** |  |  |  |  |  |  |  |  |  |
| NO | 1894 (33.03%) | 146 (30.67%) | 0.32 | 0.05 |  | 135 (28.42%) | 145 (30.53%) | 0.52 | 0.05 |
| YES | 3840 (66.97%) | 330 (69.33%) |  |  |  | 340 (71.58%) | 330 (69.47%) |  |  |
| **AFIB** |  |  |  |  |  |  |  |  |  |
| NO | 4587 (80.00%) | 368 (77.31%) | 0.18 | 0.07 |  | 376 (79.16%) | 368 (77.47%) | 0.58 | 0.04 |
| YES | 1147 (20.00%) | 108 (22.69%) |  |  |  | 99 (20.84%) | 107 (22.53%) |  |  |
| **CAD** |  |  |  |  |  |  |  |  |  |
| NO | 3615 (63.04%) | 296 (62.18%) | 0.75 | 0.02 |  | 285 (60.00%) | 295 (62.11%) | 0.55 | 0.04 |
| YES | 2119 (36.96%) | 180 (37.82%) |  |  |  | 190 (40.00%) | 180 (37.89%) |  |  |
| **CHF** |  |  |  |  |  |  |  |  |  |
| NO | 3557 (62.03%) | 275 (57.77%) | 0.074 | 0.09 |  | 271 (57.05%) | 274 (57.68%) | 0.9 | 0.01 |
| YES | 2177 (37.97%) | 201 (42.23%) |  |  |  | 204 (42.95%) | 201 (42.32%) |  |  |
| **COPD** |  |  |  |  |  |  |  |  |  |
| NO | 4777 (83.31%) | 394 (82.77%) | 0.81 | 0.01 |  | 403 (84.84%) | 393 (82.74%) | 0.43 | 0.06 |
| YES | 957 (16.69%) | 82 (17.23%) |  |  |  | 72 (15.16%) | 82 (17.26%) |  |  |
| **Liver** |  |  |  |  |  |  |  |  |  |
| NO | 5292 (92.29%) | 420 (88.24%) | <0.05 | 0.14 |  | 419 (88.21%) | 419 (88.21%) | 1 | 0 |
| YES | 442 (7.71%) | 56 (11.76%) |  |  |  | 56 (11.79%) | 56 (11.79%) |  |  |
| **Malignancy** |  |  |  |  |  |  |  |  |  |
| NO | 4618 (80.54%) | 380 (79.83%) | 0.75 | 0.02 |  | 385 (81.05%) | 379 (79.79%) | 0.68 | 0.03 |
| YES | 1116 (19.46%) | 96 (20.17%) |  |  |  | 90 (18.95%) | 96 (20.21%) |  |  |
| **Renal** |  |  |  |  |  |  |  |  |  |
| NO | 4218 (73.56%) | 313 (65.76%) | <0.05 | 0.17 |  | 290 (61.05%) | 312 (65.68%) | 0.16 | 0.1 |
| YES | 1516 (26.44%) | 163 (34.24%) |  |  |  | 185 (38.95%) | 163 (34.32%) |  |  |
| **Stroke** |  |  |  |  |  |  |  |  |  |
| NO | 5334 (93.02%) | 429 (90.13%) | <0.05 | 0.1 |  | 429 (90.32%) | 428 (90.11%) | 1 | 0.01 |
| YES | 400 (6.98%) | 47 (9.87%) |  |  |  | 46 (9.68%) | 47 (9.89%) |  |  |
| **Heart rate** |  |  |  |  |  |  |  |  |  |
| Mean (SD) | 91.84 (20.89) | 93.03 (21.07) | 0.12 | 0.06 |  | 92.69 (22.02) | 93.01 (21.09) | 0.54 | 0.01 |
| **MAP** |  |  |  |  |  |  |  |  |  |
| Mean (SD) | 77.44 (18.58) | 77.57 (19.30) | 0.68 | 0.01 |  | 77.69 (19.62) | 77.56 (19.32) | 0.65 | 0.01 |
| **Temperature** |  |  |  |  |  |  |  |  |  |
| Mean (SD) | 36.67 (1.76) | 36.55 (1.12) | 0.12 | 0.08 |  | 36.51 (1.11) | 36.55 (1.12) | 0.52 | 0.04 |
| **WBC** |  |  |  |  |  |  |  |  |  |
| Mean (SD) | 14.95 (9.63) | 14.72 (8.90) | 0.87 | 0.02 |  | 14.58 (8.89) | 14.70 (8.90) | 0.35 | 0.01 |
| **Hemoglobin** |  |  |  |  |  |  |  |  |  |
| Mean (SD) | 10.06 (2.23) | 10.26 (2.36) | 0.2 | 0.09 |  | 10.13 (2.36) | 10.26 (2.36) | 0.55 | 0.05 |
| **Platelet** |  |  |  |  |  |  |  |  |  |
| Mean (SD) | 199.95 (116.51) | 188.34 (106.05) | 0.13 | 0.1 |  | 191.14 (123.64) | 188.64 (105.97) | 0.52 | 0.02 |
| **Sodium** |  |  |  |  |  |  |  |  |  |
| Mean (SD) | 137.48 (13.12) | 136.63 (5.82) | 0.1 | 0.08 |  | 136.59 (6.09) | 136.61 (5.82) | 0.78 | 0 |
| **Potassium** |  |  |  |  |  |  |  |  |  |
| Mean (SD) | 4.35 (0.90) | 4.38 (0.90) | 0.31 | 0.03 |  | 4.35 (0.88) | 4.38 (0.90) | 0.82 | 0.02 |
| **Chloride** |  |  |  |  |  |  |  |  |  |
| Mean (SD) | 104.47 (8.06) | 102.48 (8.07) | <0.05 | 0.25 |  | 102.65 (7.97) | 102.50 (8.08) | 0.64 | 0.02 |
| **Bun** |  |  |  |  |  |  |  |  |  |
| Mean (SD) | 31.38 (23.57) | 40.23 (29.48) | <0.05 | 0.33 |  | 41.60 (31.89) | 39.96 (28.92) | 0.51 | 0.05 |
| **Creatinine** |  |  |  |  |  |  |  |  |  |
| Mean (SD) | 1.74 (1.71) | 2.29 (1.94) | <0.05 | 0.3 |  | 2.37 (2.16) | 2.28 (1.94) | 0.92 | 0.04 |
| **PH** |  |  |  |  |  |  |  |  |  |
| Mean (SD) | 7.34 (0.11) | 7.32 (0.12) | <0.05 | 0.19 |  | 7.31 (0.12) | 7.32 (0.12) | 0.93 | 0.02 |
| **PO2** |  |  |  |  |  |  |  |  |  |
| Mean (SD) | 156.62 (130.28) | 134.41 (119.24) | <0.05 | 0.18 |  | 135.09 (117.90) | 134.53 (119.34) | 0.69 | 0 |
| **PCO2** |  |  |  |  |  |  |  |  |  |
| Mean (SD) | 42.83 (12.54) | 42.01 (13.01) | 0.18 | 0.06 |  | 41.48 (12.71) | 41.99 (13.02) | 0.48 | 0.04 |
| **Lactate** |  |  |  |  |  |  |  |  |  |
| Mean (SD) | 2.72 (2.19) | 3.36 (2.87) | <0.05 | 0.25 |  | 3.39 (3.10) | 3.36 (2.88) | 0.45 | 0.01 |
| **Bicarbonate** |  |  |  |  |  |  |  |  |  |
| Mean (SD) | 21.26 (4.84) | 20.36 (5.11) | <0.05 | 0.18 |  | 20.05 (5.69) | 20.34 (5.10) | 0.49 | 0.05 |
| **CVP (tested)** |  |  |  |  |  |  |  |  |  |
| NO | 2869 (50.03%) | 270 (56.72%) | <0.05 | 0.13 |  | 277 (58.32%) | 269 (56.63%) | 0.65 | 0.03 |
| YES | 2865 (49.97%) | 206 (43.28%) |  |  |  | 198 (41.68%) | 206 (43.37%) |  |  |
| **BNP (tested)** |  |  |  |  |  |  |  |  |  |
| NO | 5418 (94.49%) | 436 (91.60%) | <0.05 | 0.11 |  | 437 (92.00%) | 435 (91.58%) | 0.91 | 0.02 |
| YES | 316 (5.51%) | 40 (8.40%) |  |  |  | 38 (8.00%) | 40 (8.42%) |  |  |
| **Troponin (tested)** |  |  |  |  |  |  |  |  |  |
| NO | 3743 (65.28%) | 252 (52.94%) | <0.05 | 0.25 |  | 248 (52.21%) | 251 (52.84%) | 0.9 | 0.01 |
| YES | 1991 (34.72%) | 224 (47.06%) |  |  |  | 227 (47.79%) | 224 (47.16%) |  |  |
| **Creatinine kinase (tested)** |  |  |  |  |  |  |  |  |  |
| NO | 3608 (62.92%) | 250 (52.52%) | <0.05 | 0.21 |  | 247 (52.00%) | 249 (52.42%) | 0.95 | 0.01 |
| YES | 2126 (37.08%) | 226 (47.48%) |  |  |  | 228 (48.00%) | 226 (47.58%) |  |  |

**Supplementary Table 5. Baseline Characteristics Before and After Propensity Score Matching of Cohort 4**

|  | Before matching | | | |  | After matching | | | |
| --- | --- | --- | --- | --- | --- | --- | --- | --- | --- |
|  | 0 ≤ VRR < 50% (N=2084) | -50% ≤ VRR < 0 (N=593) | P-value | SMD |  | 0 ≤ VRR < 50% (N=592) | -50% ≤ VRR < 0 (N=592) | P-value | SMD |
| **Age** |  |  |  |  |  |  |  |  |  |
| Mean (SD) | 66.90 (15.18) | 66.42 (14.94) | 0.44 | 0.03 |  | 66.42 (15.72) | 66.45 (14.94) | 0.93 | 0 |
| **Gender** |  |  |  |  |  |  |  |  |  |
| Female | 886 (42.51%) | 254 (42.83%) | 0.93 | 0.01 |  | 255 (43.07%) | 254 (42.91%) | 1 | 0 |
| Male | 1198 (57.49%) | 339 (57.17%) |  |  |  | 337 (56.93%) | 338 (57.09%) |  |  |
| **Weight** |  |  |  |  |  |  |  |  |  |
| Mean (SD) | 83.40 (24.86) | 83.14 (25.21) | 0.57 | 0.01 |  | 83.31 (24.30) | 83.14 (25.24) | 0.73 | 0.01 |
| **SAPS II** |  |  |  |  |  |  |  |  |  |
| Mean (SD) | 50.03 (15.40) | 51.77 (14.88) | <0.05 | 0.11 |  | 50.56 (16.22) | 51.77 (14.90) | 0.088 | 0.08 |
| **SOFA score** |  |  |  |  |  |  |  |  |  |
| Mean (SD) | 10.95 (3.79) | 11.66 (3.93) | <0.05 | 0.18 |  | 11.43 (3.97) | 11.65 (3.93) | 0.32 | 0.06 |
| **First nonzero VIS** |  |  |  |  |  |  |  |  |  |
| Mean (SD) | 15.63 (15.14) | 13.16 (12.23) | <0.05 | 0.18 |  | 12.45 (11.75) | 13.15 (12.24) | 0.32 | 0.06 |
| **Mechanical ventilation** |  |  |  |  |  |  |  |  |  |
| NO | 834 (40.02%) | 233 (39.29%) | 0.79 | 0.01 |  | 250 (42.23%) | 233 (39.36%) | 0.34 | 0.06 |
| YES | 1250 (59.98%) | 360 (60.71%) |  |  |  | 342 (57.77%) | 359 (60.64%) |  |  |
| **Sedative** |  |  |  |  |  |  |  |  |  |
| NO | 560 (26.87%) | 148 (24.96%) | 0.38 | 0.04 |  | 154 (26.01%) | 148 (25.00%) | 0.74 | 0.02 |
| YES | 1524 (73.13%) | 445 (75.04%) |  |  |  | 438 (73.99%) | 444 (75.00%) |  |  |
| **AFIB** |  |  |  |  |  |  |  |  |  |
| NO | 1677 (80.47%) | 475 (80.10%) | 0.89 | 0.01 |  | 480 (81.08%) | 474 (80.07%) | 0.71 | 0.03 |
| YES | 407 (19.53%) | 118 (19.90%) |  |  |  | 112 (18.92%) | 118 (19.93%) |  |  |
| **CAD** |  |  |  |  |  |  |  |  |  |
| NO | 1377 (66.07%) | 380 (64.08%) | 0.39 | 0.04 |  | 382 (64.53%) | 379 (64.02%) | 0.9 | 0.01 |
| YES | 707 (33.93%) | 213 (35.92%) |  |  |  | 210 (35.47%) | 213 (35.98%) |  |  |
| **CHF** |  |  |  |  |  |  |  |  |  |
| NO | 1219 (58.49%) | 362 (61.05%) | 0.29 | 0.05 |  | 345 (58.28%) | 361 (60.98%) | 0.37 | 0.06 |
| YES | 865 (41.51%) | 231 (38.95%) |  |  |  | 247 (41.72%) | 231 (39.02%) |  |  |
| **COPD** |  |  |  |  |  |  |  |  |  |
| NO | 1738 (83.40%) | 477 (80.44%) | 0.11 | 0.08 |  | 466 (78.72%) | 476 (80.41%) | 0.52 | 0.04 |
| YES | 346 (16.60%) | 116 (19.56%) |  |  |  | 126 (21.28%) | 116 (19.59%) |  |  |
| **Liver** |  |  |  |  |  |  |  |  |  |
| NO | 1828 (87.72%) | 510 (86.00%) | 0.3 | 0.05 |  | 493 (83.28%) | 510 (86.15%) | 0.2 | 0.08 |
| YES | 256 (12.28%) | 83 (14.00%) |  |  |  | 99 (16.72%) | 82 (13.85%) |  |  |
| **Malignancy** |  |  |  |  |  |  |  |  |  |
| NO | 1673 (80.28%) | 478 (80.61%) | 0.91 | 0.01 |  | 482 (81.42%) | 477 (80.57%) | 0.77 | 0.02 |
| YES | 411 (19.72%) | 115 (19.39%) |  |  |  | 110 (18.58%) | 115 (19.43%) |  |  |
| **Renal** |  |  |  |  |  |  |  |  |  |
| NO | 1430 (68.62%) | 397 (66.95%) | 0.47 | 0.04 |  | 404 (68.24%) | 396 (66.89%) | 0.66 | 0.03 |
| YES | 654 (31.38%) | 196 (33.05%) |  |  |  | 188 (31.76%) | 196 (33.11%) |  |  |
| **Stroke** |  |  |  |  |  |  |  |  |  |
| NO | 1922 (92.23%) | 547 (92.24%) | 1 | 0 |  | 540 (91.22%) | 546 (92.23%) | 0.6 | 0.04 |
| YES | 162 (7.77%) | 46 (7.76%) |  |  |  | 52 (8.78%) | 46 (7.77%) |  |  |
| **Heart rate** |  |  |  |  |  |  |  |  |  |
| Mean (SD) | 94.44 (21.69) | 93.65 (20.68) | 0.69 | 0.04 |  | 92.10 (22.08) | 93.66 (20.70) | 0.11 | 0.07 |
| **MAP** |  |  |  |  |  |  |  |  |  |
| Mean (SD) | 76.26 (19.23) | 75.88 (19.27) | 0.66 | 0.02 |  | 74.89 (18.50) | 75.82 (19.23) | 0.55 | 0.05 |
| **Temperature** |  |  |  |  |  |  |  |  |  |
| Mean (SD) | 36.61 (1.12) | 36.50 (1.31) | 0.33 | 0.09 |  | 36.50 (1.20) | 36.51 (1.30) | 0.65 | 0.01 |
| **WBC** |  |  |  |  |  |  |  |  |  |
| Mean (SD) | 14.89 (10.65) | 14.78 (9.26) | 1 | 0.01 |  | 14.26 (12.20) | 14.78 (9.27) | 0.076 | 0.05 |
| **Hemoglobin** |  |  |  |  |  |  |  |  |  |
| Mean (SD) | 10.25 (2.33) | 10.24 (2.23) | 0.8 | 0 |  | 10.17 (2.32) | 10.24 (2.23) | 0.46 | 0.03 |
| **Platelet** |  |  |  |  |  |  |  |  |  |
| Mean (SD) | 201.86 (123.39) | 191.12 (120.36) | <0.05 | 0.09 |  | 188.93 (125.11) | 191.18 (120.45) | 0.67 | 0.02 |
| **Sodium** |  |  |  |  |  |  |  |  |  |
| Mean (SD) | 136.90 (6.01) | 137.08 (6.18) | 0.14 | 0.03 |  | 137.07 (6.35) | 137.06 (6.17) | 0.39 | 0 |
| **Potassium** |  |  |  |  |  |  |  |  |  |
| Mean (SD) | 4.33 (0.91) | 4.44 (0.92) | <0.05 | 0.12 |  | 4.43 (0.93) | 4.43 (0.92) | 0.79 | 0 |
| **Chloride** |  |  |  |  |  |  |  |  |  |
| Mean (SD) | 102.98 (7.79) | 102.83 (7.98) | 0.79 | 0.02 |  | 103.02 (8.11) | 102.85 (7.98) | 0.77 | 0.02 |
| **Bun** |  |  |  |  |  |  |  |  |  |
| Mean (SD) | 37.47 (26.87) | 40.25 (28.66) | <0.05 | 0.1 |  | 40.36 (30.02) | 40.29 (28.67) | 0.7 | 0 |
| **Creatinine** |  |  |  |  |  |  |  |  |  |
| Mean (SD) | 2.07 (1.83) | 2.26 (1.96) | <0.05 | 0.1 |  | 2.22 (1.89) | 2.26 (1.96) | 0.33 | 0.02 |
| **PH** |  |  |  |  |  |  |  |  |  |
| Mean (SD) | 7.32 (0.12) | 7.31 (0.12) | 0.3 | 0.05 |  | 7.32 (0.12) | 7.31 (0.12) | 0.2 | 0.07 |
| **PO2** |  |  |  |  |  |  |  |  |  |
| Mean (SD) | 129.82 (112.18) | 129.90 (112.26) | 0.61 | 0 |  | 131.53 (113.59) | 129.95 (112.35) | 0.91 | 0.01 |
| **PCO2** |  |  |  |  |  |  |  |  |  |
| Mean (SD) | 43.25 (12.95) | 42.83 (13.84) | 0.23 | 0.03 |  | 42.47 (12.79) | 42.85 (13.85) | 0.74 | 0.03 |
| **Lactate** |  |  |  |  |  |  |  |  |  |
| Mean (SD) | 3.21 (2.77) | 3.43 (2.95) | <0.05 | 0.08 |  | 3.20 (2.74) | 3.39 (2.82) | 0.14 | 0.07 |
| **Bicarbonate** |  |  |  |  |  |  |  |  |  |
| Mean (SD) | 20.55 (5.42) | 20.13 (5.45) | 0.077 | 0.08 |  | 20.33 (5.16) | 20.15 (5.43) | 0.33 | 0.03 |
| **CVP (tested)** |  |  |  |  |  |  |  |  |  |
| NO | 1058 (50.77%) | 333 (56.16%) | <0.05 | 0.11 |  | 331 (55.91%) | 332 (56.08%) | 1 | 0 |
| YES | 1026 (49.23%) | 260 (43.84%) |  |  |  | 261 (44.09%) | 260 (43.92%) |  |  |
| **BNP (tested)** |  |  |  |  |  |  |  |  |  |
| NO | 1955 (93.81%) | 549 (92.58%) | 0.33 | 0.05 |  | 542 (91.55%) | 548 (92.57%) | 0.59 | 0.04 |
| YES | 129 (6.19%) | 44 (7.42%) |  |  |  | 50 (8.45%) | 44 (7.43%) |  |  |
| **Troponin (tested)** |  |  |  |  |  |  |  |  |  |
| NO | 1210 (58.06%) | 319 (53.79%) | 0.071 | 0.09 |  | 326 (55.07%) | 319 (53.89%) | 0.73 | 0.02 |
| YES | 874 (41.94%) | 274 (46.21%) |  |  |  | 266 (44.93%) | 273 (46.11%) |  |  |
| **Creatinine kinase (tested)** |  |  |  |  |  |  |  |  |  |
| NO | 1140 (54.70%) | 302 (50.93%) | 0.11 | 0.08 |  | 304 (51.35%) | 302 (51.01%) | 0.95 | 0.01 |
| YES | 944 (45.30%) | 291 (49.07%) |  |  |  | 288 (48.65%) | 290 (48.99%) |  |  |

**Supplementary Table 6. Baseline Characteristics Before and After Propensity Score Matching of Cohort 5**

|  | Before matching | | | |  | After matching | | | |
| --- | --- | --- | --- | --- | --- | --- | --- | --- | --- |
|  | 0 ≤ VRR < 50% (N=2084) | VRR < -50% (N=476) | P-value | SMD |  | 0 ≤ VRR < 50% (N=473) | VRR < -50% (N=473) | P-value | SMD |
| **Age** |  |  |  |  |  |  |  |  |  |
| Mean (SD) | 66.90 (15.18) | 66.63 (14.62) | 0.69 | 0.02 |  | 66.89 (15.79) | 66.72 (14.57) | 0.74 | 0.01 |
| **Gender** |  |  |  |  |  |  |  |  |  |
| Female | 886 (42.51%) | 200 (42.02%) | 0.88 | 0.01 |  | 202 (42.71%) | 198 (41.86%) | 0.84 | 0.02 |
| Male | 1198 (57.49%) | 276 (57.98%) |  |  |  | 271 (57.29%) | 275 (58.14%) |  |  |
| **Weight** |  |  |  |  |  |  |  |  |  |
| Mean (SD) | 83.40 (24.86) | 83.28 (26.16) | 0.88 | 0 |  | 82.92 (23.92) | 83.40 (26.19) | 0.81 | 0.02 |
| **SAPS II** |  |  |  |  |  |  |  |  |  |
| Mean (SD) | 50.03 (15.40) | 49.67 (14.82) | 0.92 | 0.02 |  | 48.55 (15.48) | 49.62 (14.81) | 0.17 | 0.07 |
| **SOFA score** |  |  |  |  |  |  |  |  |  |
| Mean (SD) | 10.95 (3.79) | 11.13 (3.79) | 0.35 | 0.05 |  | 10.88 (3.88) | 11.09 (3.76) | 0.38 | 0.05 |
| **First nonzero VIS** |  |  |  |  |  |  |  |  |  |
| Mean (SD) | 15.63 (15.14) | 10.72 (11.92) | <0.05 | 0.36 |  | 10.71 (11.08) | 10.48 (10.59) | 0.29 | 0.02 |
| **Mechanical ventilation** |  |  |  |  |  |  |  |  |  |
| NO | 834 (40.02%) | 217 (45.59%) | <0.05 | 0.11 |  | 235 (49.68%) | 215 (45.45%) | 0.22 | 0.08 |
| YES | 1250 (59.98%) | 259 (54.41%) |  |  |  | 238 (50.32%) | 258 (54.55%) |  |  |
| **Sedative** |  |  |  |  |  |  |  |  |  |
| NO | 560 (26.87%) | 146 (30.67%) | 0.11 | 0.08 |  | 150 (31.71%) | 145 (30.66%) | 0.78 | 0.02 |
| YES | 1524 (73.13%) | 330 (69.33%) |  |  |  | 323 (68.29%) | 328 (69.34%) |  |  |
| **AFIB** |  |  |  |  |  |  |  |  |  |
| NO | 1677 (80.47%) | 368 (77.31%) | 0.14 | 0.08 |  | 369 (78.01%) | 365 (77.17%) | 0.82 | 0.02 |
| YES | 407 (19.53%) | 108 (22.69%) |  |  |  | 104 (21.99%) | 108 (22.83%) |  |  |
| **CAD** |  |  |  |  |  |  |  |  |  |
| NO | 1376 (66.03%) | 296 (62.18%) | 0.12 | 0.08 |  | 278 (58.77%) | 293 (61.95%) | 0.35 | 0.06 |
| YES | 708 (33.97%) | 180 (37.82%) |  |  |  | 195 (41.23%) | 180 (38.05%) |  |  |
| **CHF** |  |  |  |  |  |  |  |  |  |
| NO | 1220 (58.54%) | 275 (57.77%) | 0.8 | 0.02 |  | 260 (54.97%) | 273 (57.72%) | 0.43 | 0.06 |
| YES | 864 (41.46%) | 201 (42.23%) |  |  |  | 213 (45.03%) | 200 (42.28%) |  |  |
| **COPD** |  |  |  |  |  |  |  |  |  |
| NO | 1739 (83.45%) | 394 (82.77%) | 0.77 | 0.02 |  | 389 (82.24%) | 391 (82.66%) | 0.93 | 0.01 |
| YES | 345 (16.55%) | 82 (17.23%) |  |  |  | 84 (17.76%) | 82 (17.34%) |  |  |
| **Liver** |  |  |  |  |  |  |  |  |  |
| NO | 1829 (87.76%) | 420 (88.24%) | 0.84 | 0.01 |  | 404 (85.41%) | 417 (88.16%) | 0.25 | 0.08 |
| YES | 255 (12.24%) | 56 (11.76%) |  |  |  | 69 (14.59%) | 56 (11.84%) |  |  |
| **Malignancy** |  |  |  |  |  |  |  |  |  |
| NO | 1673 (80.28%) | 380 (79.83%) | 0.88 | 0.01 |  | 390 (82.45%) | 377 (79.70%) | 0.32 | 0.07 |
| YES | 411 (19.72%) | 96 (20.17%) |  |  |  | 83 (17.55%) | 96 (20.30%) |  |  |
| **Renal** |  |  |  |  |  |  |  |  |  |
| NO | 1430 (68.62%) | 313 (65.76%) | 0.25 | 0.06 |  | 314 (66.38%) | 311 (65.75%) | 0.89 | 0.01 |
| YES | 654 (31.38%) | 163 (34.24%) |  |  |  | 159 (33.62%) | 162 (34.25%) |  |  |
| **Stroke** |  |  |  |  |  |  |  |  |  |
| NO | 1922 (92.23%) | 429 (90.13%) | 0.16 | 0.07 |  | 419 (88.58%) | 426 (90.06%) | 0.53 | 0.05 |
| YES | 162 (7.77%) | 47 (9.87%) |  |  |  | 54 (11.42%) | 47 (9.94%) |  |  |
| **Heart rate** |  |  |  |  |  |  |  |  |  |
| Mean (SD) | 94.44 (21.69) | 93.03 (21.07) | 0.19 | 0.07 |  | 90.29 (21.70) | 92.94 (21.07) | 0.078 | 0.12 |
| **MAP** |  |  |  |  |  |  |  |  |  |
| Mean (SD) | 76.26 (19.23) | 77.57 (19.30) | 0.055 | 0.07 |  | 76.34 (19.73) | 77.39 (19.18) | 0.2 | 0.05 |
| **Temperature** |  |  |  |  |  |  |  |  |  |
| Mean (SD) | 36.60 (1.16) | 36.55 (1.13) | 0.24 | 0.05 |  | 36.53 (1.16) | 36.55 (1.12) | 0.89 | 0.02 |
| **WBC** |  |  |  |  |  |  |  |  |  |
| Mean (SD) | 14.88 (10.64) | 14.77 (8.92) | 0.55 | 0.01 |  | 14.32 (13.07) | 14.78 (8.93) | <0.05 | 0.04 |
| **Hemoglobin** |  |  |  |  |  |  |  |  |  |
| Mean (SD) | 10.26 (2.33) | 10.27 (2.37) | 0.98 | 0.01 |  | 10.23 (2.35) | 10.27 (2.38) | 0.93 | 0.02 |
| **Platelet** |  |  |  |  |  |  |  |  |  |
| Mean (SD) | 201.81 (123.35) | 188.62 (106.27) | 0.14 | 0.11 |  | 178.27 (103.43) | 189.30 (106.26) | 0.052 | 0.11 |
| **Sodium** |  |  |  |  |  |  |  |  |  |
| Mean (SD) | 136.90 (6.01) | 136.63 (5.82) | 0.81 | 0.05 |  | 136.66 (6.00) | 136.63 (5.80) | 0.61 | 0.01 |
| **Potassium** |  |  |  |  |  |  |  |  |  |
| Mean (SD) | 4.33 (0.91) | 4.38 (0.90) | 0.13 | 0.06 |  | 4.36 (0.89) | 4.37 (0.90) | 0.68 | 0.02 |
| **Chloride** |  |  |  |  |  |  |  |  |  |
| Mean (SD) | 102.98 (7.79) | 102.48 (8.07) | 0.29 | 0.06 |  | 102.83 (7.91) | 102.49 (8.07) | 0.58 | 0.04 |
| **Bun** |  |  |  |  |  |  |  |  |  |
| Mean (SD) | 37.46 (26.87) | 40.23 (29.48) | 0.19 | 0.1 |  | 40.25 (29.35) | 40.29 (29.56) | 0.88 | 0 |
| **Creatinine** |  |  |  |  |  |  |  |  |  |
| Mean (SD) | 2.07 (1.83) | 2.29 (1.94) | <0.05 | 0.11 |  | 2.22 (1.90) | 2.29 (1.94) | 0.57 | 0.03 |
| **PH** |  |  |  |  |  |  |  |  |  |
| Mean (SD) | 7.31 (0.12) | 7.32 (0.12) | 0.44 | 0.04 |  | 7.33 (0.11) | 7.32 (0.12) | 0.29 | 0.07 |
| **PO2** |  |  |  |  |  |  |  |  |  |
| Mean (SD) | 129.49 (111.74) | 132.99 (118.01) | 0.9 | 0.03 |  | 136.35 (114.73) | 132.71 (117.21) | 0.27 | 0.03 |
| **PCO2** |  |  |  |  |  |  |  |  |  |
| Mean (SD) | 43.39 (13.18) | 41.96 (12.81) | 0.058 | 0.11 |  | 41.14 (11.30) | 41.99 (12.82) | 0.31 | 0.07 |
| **Lactate** |  |  |  |  |  |  |  |  |  |
| Mean (SD) | 3.24 (2.77) | 3.30 (2.83) | 0.54 | 0.02 |  | 3.14 (2.63) | 3.26 (2.76) | 0.4 | 0.05 |
| **Bicarbonate** |  |  |  |  |  |  |  |  |  |
| Mean (SD) | 20.55 (5.42) | 20.36 (5.11) | 0.66 | 0.04 |  | 20.42 (4.89) | 20.41 (5.07) | 0.81 | 0 |
| **CVP (tested)** |  |  |  |  |  |  |  |  |  |
| NO | 1058 (50.77%) | 270 (56.72%) | <0.05 | 0.12 |  | 270 (57.08%) | 268 (56.66%) | 0.95 | 0.01 |
| YES | 1026 (49.23%) | 206 (43.28%) |  |  |  | 203 (42.92%) | 205 (43.34%) |  |  |
| **BNP (tested)** |  |  |  |  |  |  |  |  |  |
| NO | 1955 (93.81%) | 436 (91.60%) | 0.098 | 0.09 |  | 431 (91.12%) | 434 (91.75%) | 0.82 | 0.02 |
| YES | 129 (6.19%) | 40 (8.40%) |  |  |  | 42 (8.88%) | 39 (8.25%) |  |  |
| **Troponin (tested)** |  |  |  |  |  |  |  |  |  |
| NO | 1210 (58.06%) | 252 (52.94%) | <0.05 | 0.1 |  | 260 (54.97%) | 251 (53.07%) | 0.6 | 0.04 |
| YES | 874 (41.94%) | 224 (47.06%) |  |  |  | 213 (45.03%) | 222 (46.93%) |  |  |
| **Creatinine kinase (tested)** |  |  |  |  |  |  |  |  |  |
| NO | 1140 (54.70%) | 250 (52.52%) | 0.42 | 0.04 |  | 257 (54.33%) | 249 (52.64%) | 0.65 | 0.03 |
| YES | 944 (45.30%) | 226 (47.48%) |  |  |  | 216 (45.67%) | 224 (47.36%) |  |  |

**Supplementary Table 7. Baseline Characteristics Before and After Propensity Score Matching of Cohort 6**

|  | Before matching | | | |  | After matching | | | |
| --- | --- | --- | --- | --- | --- | --- | --- | --- | --- |
|  | -50% ≤ VRR < 0 (N=593) | VRR < -50% (N=476) | P-value | SMD |  | -50% ≤ VRR < 0 (N=452) | VRR < -50% (N=452) | P-value | SMD |
| **Age** |  |  |  |  |  |  |  |  |  |
| Mean (SD) | 66.42 (14.94) | 66.63 (14.62) | 0.8 | 0.01 |  | 66.83 (14.70) | 66.69 (14.56) | 0.93 | 0.01 |
| **Gender** |  |  |  |  |  |  |  |  |  |
| Female | 254 (42.83%) | 200 (42.02%) | 0.84 | 0.02 |  | 196 (43.36%) | 189 (41.81%) | 0.69 | 0.03 |
| Male | 339 (57.17%) | 276 (57.98%) |  |  |  | 256 (56.64%) | 263 (58.19%) |  |  |
| **Weight** |  |  |  |  |  |  |  |  |  |
| Mean (SD) | 83.14 (25.21) | 83.28 (26.16) | 0.74 | 0.01 |  | 82.19 (22.35) | 83.16 (26.17) | 0.71 | 0.04 |
| **SAPS II** |  |  |  |  |  |  |  |  |  |
| Mean (SD) | 51.77 (14.88) | 49.67 (14.82) | <0.05 | 0.14 |  | 50.05 (14.54) | 50.12 (14.86) | 0.99 | 0 |
| **SOFA score** |  |  |  |  |  |  |  |  |  |
| Mean (SD) | 11.66 (3.93) | 11.13 (3.79) | <0.05 | 0.14 |  | 11.10 (3.73) | 11.22 (3.81) | 0.75 | 0.03 |
| **First nonzero VIS** |  |  |  |  |  |  |  |  |  |
| Mean (SD) | 13.16 (12.23) | 10.72 (11.92) | <0.05 | 0.2 |  | 11.22 (9.87) | 10.70 (10.76) | <0.05 | 0.05 |
| **Mechanical ventilation** |  |  |  |  |  |  |  |  |  |
| NO | 233 (39.29%) | 217 (45.59%) | <0.05 | 0.13 |  | 199 (44.03%) | 201 (44.47%) | 0.95 | 0.01 |
| YES | 360 (60.71%) | 259 (54.41%) |  |  |  | 253 (55.97%) | 251 (55.53%) |  |  |
| **Sedative** |  |  |  |  |  |  |  |  |  |
| NO | 148 (24.96%) | 146 (30.67%) | <0.05 | 0.13 |  | 136 (30.09%) | 134 (29.65%) | 0.94 | 0.01 |
| YES | 445 (75.04%) | 330 (69.33%) |  |  |  | 316 (69.91%) | 318 (70.35%) |  |  |
| **AFIB** |  |  |  |  |  |  |  |  |  |
| NO | 475 (80.10%) | 368 (77.31%) | 0.3 | 0.07 |  | 351 (77.65%) | 352 (77.88%) | 1 | 0.01 |
| YES | 118 (19.90%) | 108 (22.69%) |  |  |  | 101 (22.35%) | 100 (22.12%) |  |  |
| **CAD** |  |  |  |  |  |  |  |  |  |
| NO | 380 (64.08%) | 296 (62.18%) | 0.57 | 0.04 |  | 276 (61.06%) | 282 (62.39%) | 0.73 | 0.03 |
| YES | 213 (35.92%) | 180 (37.82%) |  |  |  | 176 (38.94%) | 170 (37.61%) |  |  |
| **CHF** |  |  |  |  |  |  |  |  |  |
| NO | 362 (61.05%) | 275 (57.77%) | 0.31 | 0.07 |  | 264 (58.41%) | 263 (58.19%) | 1 | 0 |
| YES | 231 (38.95%) | 201 (42.23%) |  |  |  | 188 (41.59%) | 189 (41.81%) |  |  |
| **COPD** |  |  |  |  |  |  |  |  |  |
| NO | 477 (80.44%) | 394 (82.77%) | 0.37 | 0.06 |  | 377 (83.41%) | 371 (82.08%) | 0.66 | 0.04 |
| YES | 116 (19.56%) | 82 (17.23%) |  |  |  | 75 (16.59%) | 81 (17.92%) |  |  |
| **Liver** |  |  |  |  |  |  |  |  |  |
| NO | 510 (86.00%) | 420 (88.24%) | 0.32 | 0.07 |  | 404 (89.38%) | 396 (87.61%) | 0.47 | 0.06 |
| YES | 83 (14.00%) | 56 (11.76%) |  |  |  | 48 (10.62%) | 56 (12.39%) |  |  |
| **Malignancy** |  |  |  |  |  |  |  |  |  |
| NO | 478 (80.61%) | 380 (79.83%) | 0.81 | 0.02 |  | 364 (80.53%) | 358 (79.20%) | 0.68 | 0.03 |
| YES | 115 (19.39%) | 96 (20.17%) |  |  |  | 88 (19.47%) | 94 (20.80%) |  |  |
| **Renal** |  |  |  |  |  |  |  |  |  |
| NO | 397 (66.95%) | 313 (65.76%) | 0.73 | 0.03 |  | 300 (66.37%) | 298 (65.93%) | 0.94 | 0.01 |
| YES | 196 (33.05%) | 163 (34.24%) |  |  |  | 152 (33.63%) | 154 (34.07%) |  |  |
| **Stroke** |  |  |  |  |  |  |  |  |  |
| NO | 547 (92.24%) | 429 (90.13%) | 0.27 | 0.07 |  | 410 (90.71%) | 410 (90.71%) | 1 | 0 |
| YES | 46 (7.76%) | 47 (9.87%) |  |  |  | 42 (9.29%) | 42 (9.29%) |  |  |
| **Heart rate** |  |  |  |  |  |  |  |  |  |
| Mean (SD) | 93.65 (20.68) | 93.03 (21.07) | 0.42 | 0.03 |  | 92.70 (20.82) | 92.81 (21.33) | 0.82 | 0 |
| **MAP** |  |  |  |  |  |  |  |  |  |
| Mean (SD) | 75.88 (19.27) | 77.57 (19.30) | 0.051 | 0.09 |  | 77.16 (19.63) | 77.22 (18.89) | 0.39 | 0 |
| **Temperature** |  |  |  |  |  |  |  |  |  |
| Mean (SD) | 36.50 (1.30) | 36.55 (1.17) | 0.82 | 0.04 |  | 36.52 (1.22) | 36.54 (1.20) | 0.95 | 0.02 |
| **WBC** |  |  |  |  |  |  |  |  |  |
| Mean (SD) | 14.77 (9.28) | 14.72 (8.90) | 0.69 | 0.01 |  | 14.86 (9.34) | 14.77 (8.95) | 0.7 | 0.01 |
| **Hemoglobin** |  |  |  |  |  |  |  |  |  |
| Mean (SD) | 10.24 (2.23) | 10.26 (2.36) | 0.85 | 0.01 |  | 10.29 (2.18) | 10.27 (2.38) | 0.63 | 0.01 |
| **Platelet** |  |  |  |  |  |  |  |  |  |
| Mean (SD) | 191.04 (120.44) | 188.01 (106.09) | 0.77 | 0.03 |  | 189.33 (115.72) | 188.77 (107.77) | 0.67 | 0.01 |
| **Sodium** |  |  |  |  |  |  |  |  |  |
| Mean (SD) | 137.11 (6.17) | 136.63 (5.82) | 0.14 | 0.08 |  | 136.69 (6.07) | 136.68 (5.87) | 0.83 | 0 |
| **Potassium** |  |  |  |  |  |  |  |  |  |
| Mean (SD) | 4.44 (0.92) | 4.38 (0.90) | 0.26 | 0.07 |  | 4.37 (0.85) | 4.39 (0.88) | 0.82 | 0.02 |
| **Chloride** |  |  |  |  |  |  |  |  |  |
| Mean (SD) | 102.85 (7.99) | 102.48 (8.07) | 0.48 | 0.05 |  | 102.60 (8.01) | 102.51 (8.05) | 0.8 | 0.01 |
| **Bun** |  |  |  |  |  |  |  |  |  |
| Mean (SD) | 40.25 (28.66) | 40.23 (29.48) | 0.67 | 0 |  | 41.20 (30.10) | 40.08 (29.40) | 0.51 | 0.04 |
| **Creatinine** |  |  |  |  |  |  |  |  |  |
| Mean (SD) | 2.26 (1.96) | 2.29 (1.94) | 0.83 | 0.01 |  | 2.27 (2.01) | 2.26 (1.86) | 0.85 | 0.01 |
| **PH** |  |  |  |  |  |  |  |  |  |
| Mean (SD) | 7.31 (0.12) | 7.32 (0.12) | 0.28 | 0.06 |  | 7.32 (0.12) | 7.32 (0.12) | 0.79 | 0.01 |
| **PO2** |  |  |  |  |  |  |  |  |  |
| Mean (SD) | 128.09 (111.00) | 132.12 (117.36) | 0.7 | 0.04 |  | 130.68 (113.74) | 132.32 (117.53) | 0.58 | 0.01 |
| **PCO2** |  |  |  |  |  |  |  |  |  |
| Mean (SD) | 42.64 (13.76) | 42.07 (12.96) | 0.96 | 0.04 |  | 42.10 (13.38) | 42.23 (13.02) | 0.54 | 0.01 |
| **Lactate** |  |  |  |  |  |  |  |  |  |
| Mean (SD) | 3.42 (2.93) | 3.35 (2.83) | 0.79 | 0.02 |  | 3.29 (2.66) | 3.33 (2.83) | 0.82 | 0.02 |
| **Bicarbonate** |  |  |  |  |  |  |  |  |  |
| Mean (SD) | 20.15 (5.45) | 20.36 (5.11) | 0.36 | 0.04 |  | 20.26 (5.24) | 20.36 (5.07) | 0.54 | 0.02 |
| **CVP (tested)** |  |  |  |  |  |  |  |  |  |
| NO | 333 (56.16%) | 270 (56.72%) | 0.9 | 0.01 |  | 254 (56.19%) | 254 (56.19%) | 1 | 0 |
| YES | 260 (43.84%) | 206 (43.28%) |  |  |  | 198 (43.81%) | 198 (43.81%) |  |  |
| **BNP (tested)** |  |  |  |  |  |  |  |  |  |
| NO | 549 (92.58%) | 436 (91.60%) | 0.63 | 0.04 |  | 416 (92.04%) | 417 (92.26%) | 1 | 0.01 |
| YES | 44 (7.42%) | 40 (8.40%) |  |  |  | 36 (7.96%) | 35 (7.74%) |  |  |
| **Troponin (tested)** |  |  |  |  |  |  |  |  |  |
| NO | 319 (53.79%) | 252 (52.94%) | 0.83 | 0.02 |  | 235 (51.99%) | 242 (53.54%) | 0.69 | 0.03 |
| YES | 274 (46.21%) | 224 (47.06%) |  |  |  | 217 (48.01%) | 210 (46.46%) |  |  |
| **Creatinine kinase (tested)** |  |  |  |  |  |  |  |  |  |
| NO | 302 (50.93%) | 250 (52.52%) | 0.65 | 0.03 |  | 228 (50.44%) | 236 (52.21%) | 0.64 | 0.04 |
| YES | 291 (49.07%) | 226 (47.48%) |  |  |  | 224 (49.56%) | 216 (47.79%) |  |  |

**Supplementary Table 8. Pairwise comparisons between groups for ICU mortality survival curves of original cohorts**

| Group | 50% ≤ VRR | 0 ≤ VRR < 50% | -50% ≤ VRR < 0 |
| --- | --- | --- | --- |
| 0 ≤ VRR < 50% | 4.44e-19 | - | - |
| -50% ≤ VRR < 0 | 7.77e-27 | 3.59e-05 | - |
| VRR < -50% | 4.07e-31 | 4.37e-07 | 0.29 |

**Supplementary Table 9. Multivariate Cox regression analysis for ICU mortality of original cohorts**

| Characteristic | HR^1^ | 95% CI^1^ | p-value |
| --- | --- | --- | --- |
| Group |  |  |  |
| 50% ≤ VRR | — | — |  |
| 0 ≤ VRR < 50% | 1.67 | 1.50, 1.87 | <0.001 |
| -50% ≤ VRR < 0 | 2.25 | 1.95, 2.61 | <0.001 |
| VRR < -50% | 2.52 | 2.15, 2.96 | <0.001 |
| Gender |  |  |  |
| Female | — | — |  |
| Male | 0.90 | 0.82, 1.00 | 0.043 |
| Weight | 1.00 | 0.99, 1.00 | <0.001 |
| Liver |  |  |  |
| NO | — | — |  |
| YES | 1.26 | 1.09, 1.46 | 0.002 |
| Renal |  |  |  |
| NO | — | — |  |
| YES | 1.17 | 1.05, 1.29 | 0.004 |
| Heart rate | 1.00 | 1.00, 1.00 | 0.015 |
| WBC | 1.01 | 1.00, 1.01 | <0.001 |
| Platelet | 1.00 | 1.00, 1.00 | 0.042 |
| Potassium | 1.07 | 1.02, 1.13 | 0.006 |
| PCO2 | 1.01 | 1.01, 1.01 | <0.001 |

^1^HR = Hazard Ratio, CI = Confidence Interval

**Supplementary Table 10. Multivariate Cox regression analysis for ICU mortality of matched cohort 1**

| Characteristic | HR^1^ | 95% CI^1^ | p-value |
| --- | --- | --- | --- |
| Group |  |  |  |
| 50% ≤ VRR | — | — |  |
| 0 ≤ VRR < 50% | 1.32 | 1.17, 1.50 | <0.001 |
| Gender |  |  |  |
| Female | — | — |  |
| Male | 0.85 | 0.75, 0.96 | 0.012 |
| COPD |  |  |  |
| NO | — | — |  |
| YES | 1.18 | 1.01, 1.38 | 0.039 |
| Liver |  |  |  |
| NO | — | — |  |
| YES | 1.21 | 1.01, 1.46 | 0.040 |
| Malignancy |  |  |  |
| NO | — | — |  |
| YES | 1.22 | 1.05, 1.42 | 0.011 |
| Renal |  |  |  |
| NO | — | — |  |
| YES | 1.14 | 1.00, 1.31 | 0.050 |
| Heart rate | 1.00 | 1.00, 1.00 | 0.4 |
| WBC | 1.00 | 1.00, 1.01 | 0.026 |
| Potassium | 1.05 | 0.98, 1.12 | 0.2 |
| PCO2 | 1.01 | 1.00, 1.01 | 0.002 |
| ^1^HR = Hazard Ratio, CI = Confidence Interval | | | |

**Supplementary Table 11. Multivariate Cox regression analysis for ICU mortality of matched cohort 2**

| Characteristic | HR^1^ | 95% CI^1^ | p-value |
| --- | --- | --- | --- |
| Group |  |  |  |
| 50% ≤ VRR | — | — |  |
| -50% ≤ VRR < 0 | 1.79 | 1.44, 2.22 | <0.001 |
| Gender |  |  |  |
| Female | — | — |  |
| Male | 1.06 | 0.86, 1.31 | 0.6 |
| Weight | 1.00 | 0.99, 1.00 | 0.3 |
| First nonzero VIS | 1.02 | 1.01, 1.03 | <0.001 |
| Liver |  |  |  |
| NO | — | — |  |
| YES | 1.26 | 0.95, 1.67 | 0.11 |
| Malignancy |  |  |  |
| NO | — | — |  |
| YES | 1.66 | 1.31, 2.11 | <0.001 |
| Potassium | 1.19 | 1.07, 1.33 | 0.001 |
| PCO2 | 1.00 | 1.00, 1.01 | 0.4 |
| Bicarbonate | 0.98 | 0.96, 1.00 | 0.035 |
| ^1^HR = Hazard Ratio, CI = Confidence Interval | | | |

Supplementary Table 12. Multivariate Cox regression analysis for ICU mortality of matched cohort 3

| Characteristic | HR^1^ | 95% CI^1^ | p-value |
| --- | --- | --- | --- |
| Group |  |  |  |
| 50% ≤ VRR | — | — |  |
| VRR < -50% | 2.07 | 1.61, 2.66 | <0.001 |
| Gender |  |  |  |
| Female | — | — |  |
| Male | 0.99 | 0.78, 1.25 | >0.9 |
| Weight | 1.00 | 0.99, 1.00 | 0.8 |
| First nonzero VIS | 1.01 | 1.00, 1.02 | 0.041 |
| Mechanical ventilation |  |  |  |
| NO | — | — |  |
| YES | 0.98 | 0.77, 1.24 | 0.8 |
| Renal |  |  |  |
| NO | — | — |  |
| YES | 1.16 | 0.89, 1.51 | 0.3 |
| WBC | 1.00 | 0.99, 1.02 | 0.5 |
| Creatinine | 1.00 | 0.94, 1.07 | >0.9 |
| PCO2 | 1.01 | 1.00, 1.02 | 0.018 |
| Bicarbonate | 0.96 | 0.94, 0.99 | 0.004 |
| ^1^HR = Hazard Ratio, CI = Confidence Interval | | | |

**Supplementary Table 13. Multivariate Cox regression analysis for ICU mortality of matched cohort 4**

| Characteristic | HR^1^ | 95% CI^1^ | p-value |
| --- | --- | --- | --- |
| Group |  |  |  |
| 0 ≤ VRR < 50% | — | — |  |
| -50% ≤ VRR < 0 | 1.41 | 1.17, 1.70 | <0.001 |
| Weight | 1.00 | 0.99, 1.00 | 0.2 |
| CHF |  |  |  |
| NO | — | — |  |
| YES | 0.89 | 0.73, 1.10 | 0.3 |
| Liver |  |  |  |
| NO | — | — |  |
| YES | 1.41 | 1.10, 1.82 | 0.007 |
| Malignancy |  |  |  |
| NO | — | — |  |
| YES | 1.45 | 1.16, 1.81 | 0.001 |
| MAP | 1.00 | 0.99, 1.00 | 0.095 |
| Potassium | 1.14 | 1.04, 1.26 | 0.005 |
| Chloride | 1.00 | 0.98, 1.01 | 0.5 |
| PCO2 | 1.01 | 1.00, 1.02 | 0.016 |
| CVP (tested) |  |  |  |
| NO | — | — |  |
| YES | 0.95 | 0.79, 1.15 | 0.6 |
| ^1^HR = Hazard Ratio, CI = Confidence Interval | | | |

**Supplementary Table 14. Multivariate Cox regression analysis for ICU mortality of matched cohort 5**

| Characteristic | HR^1^ | 95% CI^1^ | p-value |
| --- | --- | --- | --- |
| Group |  |  |  |
| 0 ≤ VRR < 50% | — | — |  |
| VRR < -50% | 1.66 | 1.34, 2.06 | <0.001 |
| Weight | 1.00 | 1.00, 1.00 | >0.9 |
| Sedative |  |  |  |
| NO | — | — |  |
| YES | 0.93 | 0.72, 1.19 | 0.5 |
| COPD |  |  |  |
| NO | — | — |  |
| YES | 1.46 | 1.11, 1.91 | 0.006 |
| Liver |  |  |  |
| NO | — | — |  |
| YES | 1.41 | 1.06, 1.89 | 0.020 |
| Malignancy |  |  |  |
| NO | — | — |  |
| YES | 1.37 | 1.06, 1.75 | 0.015 |
| Renal |  |  |  |
| NO | — | — |  |
| YES | 1.02 | 0.80, 1.29 | 0.9 |
| MAP | 1.00 | 0.99, 1.00 | 0.3 |
| Potassium | 1.03 | 0.92, 1.17 | 0.6 |
| Chloride | 1.00 | 0.98, 1.01 | 0.8 |
| PCO2 | 1.01 | 1.00, 1.02 | 0.029 |
| CVP (tested) |  |  |  |
| NO | — | — |  |
| YES | 0.93 | 0.75, 1.16 | 0.5 |
| ^1^HR = Hazard Ratio, CI = Confidence Interval | | | |

**Supplementary Table 15. Multivariate Cox regression analysis for ICU mortality of matched cohort 6**

| Characteristic | HR^1^ | 95% CI^1^ | p-value |
| --- | --- | --- | --- |
| Group |  |  |  |
| -50% ≤ VRR < 0 | — | — |  |
| VRR < -50% | 1.23 | 1.01, 1.50 | 0.041 |
| COPD |  |  |  |
| NO | — | — |  |
| YES | 1.67 | 1.29, 2.15 | <0.001 |
| Malignancy |  |  |  |
| NO | — | — |  |
| YES | 1.54 | 1.21, 1.95 | <0.001 |
| Temperature | 0.87 | 0.81, 0.94 | <0.001 |
| Platelet | 1.00 | 1.00, 1.00 | 0.12 |
| Potassium | 1.09 | 0.97, 1.22 | 0.14 |
| Chloride | 0.99 | 0.97, 1.00 | 0.035 |
| Creatinine | 1.02 | 0.97, 1.08 | 0.4 |
| Bicarbonate | 0.97 | 0.94, 0.99 | <0.001 |
| ^1^HR = Hazard Ratio, CI = Confidence Interval | | | |

**Supplementary Table 16. Pairwise comparisons between groups for in-hospital mortality survival curves of original cohorts**

| Group | 50% ≤ VRR | 0 ≤ VRR < 50% | -50% ≤ VRR < 0 |
| --- | --- | --- | --- |
| 0 ≤ VRR < 50% | 1.21e-44 | - | - |
| -50% ≤ VRR < 0 | 3.07e-56 | 1.34e-07 | - |
| VRR < -50% | 1.53e-51 | 1.12e-07 | 0.73 |

**Supplementary Table 17. Multivariate Cox regression analysis for in-hospital mortality of original cohorts**

| Characteristic | HR^1^ | 95% CI^1^ | p-value |
| --- | --- | --- | --- |
| Group |  |  |  |
| 50% ≤ VRR | — | — |  |
| 0 ≤ VRR < 50% | 2.00 | 1.82, 2.19 | <0.001 |
| -50% ≤ VRR < 0 | 2.81 | 2.47, 3.20 | <0.001 |
| VRR < -50% | 2.91 | 2.52, 3.35 | <0.001 |
| Gender |  |  |  |
| Female | — | — |  |
| Male | 0.87 | 0.80, 0.95 | 0.001 |
| Stroke |  |  |  |
| NO | — | — |  |
| YES | 1.20 | 1.05, 1.37 | 0.009 |
| Platelet | 1.00 | 1.00, 1.00 | 0.024 |
| Potassium | 1.07 | 1.02, 1.11 | 0.005 |
| ^1^HR = Hazard Ratio, CI = Confidence Interval | | | |

**Supplementary Table 18. Multivariate Cox regression analysis for in-hospital mortality of matched cohort 1**

| Characteristic | HR^1^ | 95% CI^1^ | p-value |
| --- | --- | --- | --- |
| Group |  |  |  |
| 50% ≤ VRR | — | — |  |
| 0 ≤ VRR < 50% | 1.43 | 1.28, 1.60 | <0.001 |
| Renal |  |  |  |
| NO | — | — |  |
| YES | 1.16 | 1.03, 1.30 | 0.013 |
| Stroke |  |  |  |
| NO | — | — |  |
| YES | 1.15 | 0.96, 1.38 | 0.12 |
| WBC | 1.01 | 1.00, 1.01 | 0.002 |
| CVP (tested) |  |  |  |
| NO | — | — |  |
| YES | 0.88 | 0.79, 0.98 | 0.025 |
| ^1^HR = Hazard Ratio, CI = Confidence Interval | | | |

**Supplementary Table 19. Multivariate Cox regression analysis for in-hospital mortality of matched cohort 2**

| Characteristic | HR^1^ | 95% CI^1^ | p-value |
| --- | --- | --- | --- |
| Group |  |  |  |
| 50% ≤ VRR | — | — |  |
| -50% ≤ VRR < 0 | 1.75 | 1.45, 2.11 | <0.001 |
| Weight | 1.00 | 0.99, 1.00 | 0.2 |
| Mechanical ventilation |  |  |  |
| NO | — | — |  |
| YES | 1.18 | 0.97, 1.44 | 0.089 |
| Renal |  |  |  |
| NO | — | — |  |
| YES | 1.03 | 0.83, 1.27 | 0.8 |
| Stroke |  |  |  |
| NO | — | — |  |
| YES | 1.08 | 0.79, 1.47 | 0.6 |
| Heart rate | 1.00 | 1.00, 1.01 | 0.5 |
| Creatinine | 1.02 | 0.98, 1.07 | 0.3 |
| Bicarbonate | 0.99 | 0.97, 1.00 | 0.14 |
| CVP (tested) |  |  |  |
| NO | — | — |  |
| YES | 0.94 | 0.78, 1.13 | 0.5 |
| ^1^HR = Hazard Ratio, CI = Confidence Interval | | | |

**Supplementary Table 20. Multivariate Cox regression analysis for in-hospital mortality of matched cohort 3**

| Characteristic | HR^1^ | 95% CI^1^ | p-value |
| --- | --- | --- | --- |
| Group |  |  |  |
| 50% ≤ VRR | — | — |  |
| VRR < -50% | 2.00 | 1.61, 2.49 | <0.001 |
| Weight | 1.00 | 0.99, 1.00 | 0.2 |
| Mechanical ventilation |  |  |  |
| NO | — | — |  |
| YES | 1.12 | 0.90, 1.38 | 0.3 |
| CHF |  |  |  |
| NO | — | — |  |
| YES | 0.98 | 0.79, 1.23 | 0.9 |
| Liver |  |  |  |
| NO | — | — |  |
| YES | 1.15 | 0.85, 1.56 | 0.4 |
| Renal |  |  |  |
| NO | — | — |  |
| YES | 1.22 | 0.95, 1.55 | 0.11 |
| Stroke |  |  |  |
| NO | — | — |  |
| YES | 1.21 | 0.90, 1.64 | 0.2 |
| Heart rate | 1.00 | 1.00, 1.01 | 0.2 |
| Hemoglobin | 1.02 | 0.98, 1.07 | 0.4 |
| Creatinine | 1.00 | 0.94, 1.05 | 0.9 |
| Bicarbonate | 0.98 | 0.96, 1.00 | 0.13 |
| CVP (tested) |  |  |  |
| NO | — | — |  |
| YES | 0.93 | 0.75, 1.15 | 0.5 |
| ^1^HR = Hazard Ratio, CI = Confidence Interval | | | |

**Supplementary Table 21. Multivariate Cox regression analysis for in-hospital mortality of matched cohort 4**

| Characteristic | HR^1^ | 95% CI^1^ | p-value |
| --- | --- | --- | --- |
| Group |  |  |  |
| 0 ≤ VRR < 50% | — | — |  |
| -50% ≤ VRR < 0 | 1.48 | 1.24, 1.75 | <0.001 |
| Weight | 1.00 | 1.00, 1.00 | 0.8 |
| Mechanical ventilation |  |  |  |
| NO | — | — |  |
| YES | 1.10 | 0.92, 1.32 | 0.3 |
| CHF |  |  |  |
| NO | — | — |  |
| YES | 0.83 | 0.69, 1.00 | 0.051 |
| COPD |  |  |  |
| NO | — | — |  |
| YES | 1.15 | 0.93, 1.42 | 0.2 |
| Liver |  |  |  |
| NO | — | — |  |
| YES | 1.27 | 1.00, 1.62 | 0.051 |
| Malignancy |  |  |  |
| NO | — | — |  |
| YES | 1.53 | 1.24, 1.88 | <0.001 |
| Heart rate | 1.00 | 1.00, 1.00 | 0.8 |
| MAP | 1.00 | 0.99, 1.00 | 0.067 |
| Hemoglobin | 0.97 | 0.94, 1.01 | 0.2 |
| Platelet | 1.00 | 1.00, 1.00 | 0.036 |
| Sodium | 1.01 | 1.00, 1.03 | 0.10 |
| Potassium | 1.14 | 1.04, 1.25 | 0.005 |
| PCO2 | 1.01 | 1.00, 1.02 | 0.011 |
| CVP (tested) |  |  |  |
| NO | — | — |  |
| YES | 0.99 | 0.83, 1.18 | 0.9 |
| ^1^HR = Hazard Ratio, CI = Confidence Interval | | | |

**Supplementary Table 22. Multivariate Cox regression analysis for in-hospital mortality of matched cohort 5**

| Characteristic | HR^1^ | 95% CI^1^ | p-value |
| --- | --- | --- | --- |
| Group |  |  |  |
| 0 ≤ VRR < 50% | — | — |  |
| VRR < -50% | 1.67 | 1.37, 2.03 | <0.001 |
| Weight | 1.00 | 1.00, 1.00 | >0.9 |
| AFIB |  |  |  |
| NO | — | — |  |
| YES | 1.31 | 1.04, 1.66 | 0.024 |
| CHF |  |  |  |
| NO | — | — |  |
| YES | 0.87 | 0.71, 1.08 | 0.2 |
| COPD |  |  |  |
| NO | — | — |  |
| YES | 1.51 | 1.17, 1.95 | 0.001 |
| Liver |  |  |  |
| NO | — | — |  |
| YES | 1.05 | 0.80, 1.40 | 0.7 |
| Malignancy |  |  |  |
| NO | — | — |  |
| YES | 1.38 | 1.09, 1.73 | 0.007 |
| Heart rate | 1.00 | 1.00, 1.01 | 0.4 |
| MAP | 1.00 | 0.99, 1.00 | 0.2 |
| Platelet | 1.00 | 1.00, 1.00 | <0.001 |
| Sodium | 1.01 | 0.99, 1.03 | 0.3 |
| PCO2 | 1.01 | 1.00, 1.02 | 0.046 |
| CVP (tested) |  |  |  |
| NO | — | — |  |
| YES | 0.93 | 0.76, 1.14 | 0.5 |
| ^1^HR = Hazard Ratio, CI = Confidence Interval | | | |

**Supplementary Table 23. Multivariate Cox regression analysis for in-hospital mortality of matched cohort 6**

| Characteristic | HR^1^ | 95% CI^1^ | p-value |
| --- | --- | --- | --- |
| Group |  |  |  |
| -50% ≤ VRR < 0 | — | — |  |
| VRR < -50% | 1.16 | 0.96, 1.39 | 0.12 |
| COPD |  |  |  |
| NO | — | — |  |
| YES | 1.37 | 1.07, 1.76 | 0.012 |
| Malignancy |  |  |  |
| NO | — | — |  |
| YES | 1.45 | 1.16, 1.81 | <0.001 |
| Temperature | 0.90 | 0.84, 0.96 | 0.003 |
| WBC | 1.01 | 1.00, 1.02 | 0.087 |
| Platelet | 1.00 | 1.00, 1.00 | 0.008 |
| Chloride | 0.99 | 0.98, 1.00 | 0.14 |
| Creatinine | 1.00 | 0.95, 1.06 | >0.9 |
| PO2 | 1.00 | 1.00, 1.00 | 0.13 |
| PCO2 | 1.01 | 1.00, 1.02 | 0.048 |
| Bicarbonate | 0.97 | 0.95, 0.99 | 0.006 |
| Troponin (tested) |  |  |  |
| NO | — | — |  |
| YES | 1.23 | 0.89, 1.70 | 0.2 |
| Creatinine kinase (tested) |  |  |  |
| NO | — | — |  |
| YES | 1.13 | 0.82, 1.55 | 0.5 |
| ^1^HR = Hazard Ratio, CI = Confidence Interval | | | |
