# Supplementary figures and images for: Tendency of dynamic vasoactive and inotropic medications data as a robust predictor of mortality in patients with septic shock: An analysis of the MIMIC-IV database
Source: Front Cardiovasc Med. 2023 Mar 7;10:1126888. doi: 10.3389/fcvm.2023.1126888 (PMC10112491; doi:10.3389/fcvm.2023.1126888)

## The average impact of the input features on predictions

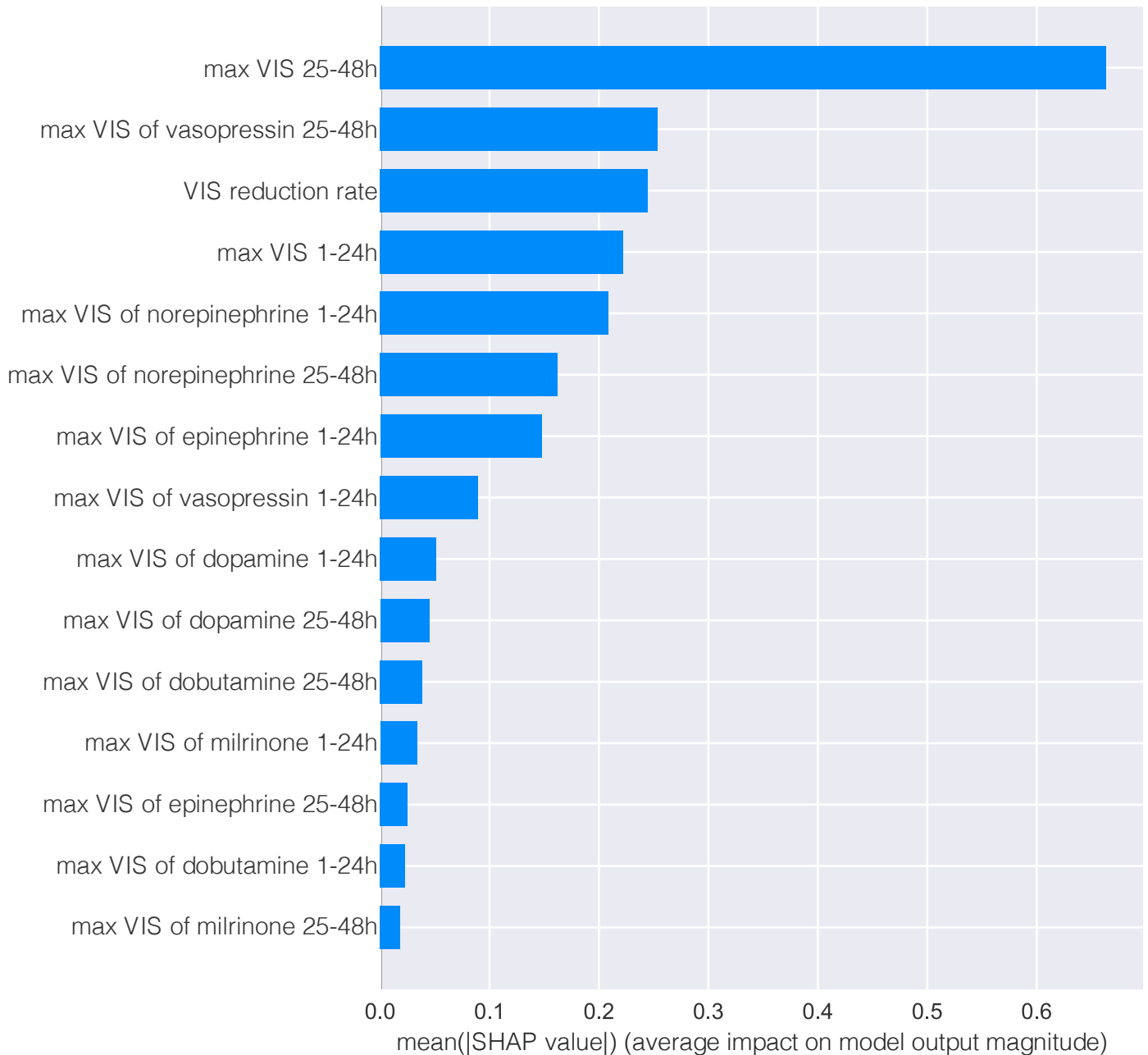

Supplement: Supplementary file 2 [file Image_1.pdf]
