# Supplementary material for: Tendency of dynamic vasoactive and inotropic medications data as a robust predictor of mortality in patients with septic shock: An analysis of the MIMIC-IV database
Source: Front Cardiovasc Med. 2023 Mar 7;10:1126888. doi: 10.3389/fcvm.2023.1126888 (PMC10112491; doi:10.3389/fcvm.2023.1126888)
Supplement: Supplementary file 3 [file Image_2.PDF]

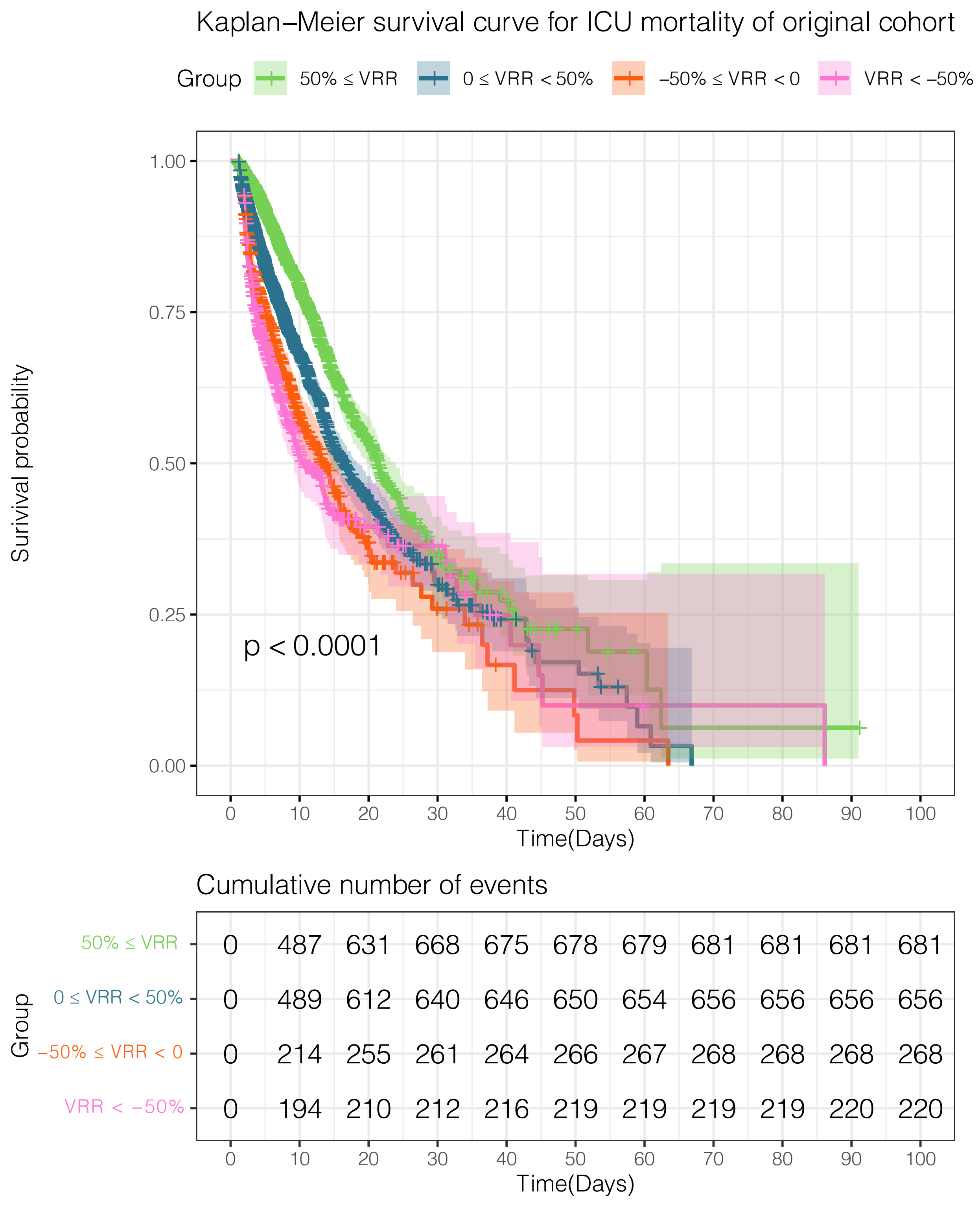
Kaplan–Meier survival curve for ICU mortality of original cohort
Group
50% ≤ VRR
0 ≤ VRR < 50%
–50% ≤ VRR < 0
VRR < –50%
Surivival probability
1.00
0.75
0.50
0.25
0.00
p < 0.0001
0
10
20
30
40
50
60
70
80
90
100
Time(Days)
Cumulative number of events
Group
50% ≤ VRR 0 487 631 668 675 678 679 681 681 681 681
0 ≤ VRR < 50% 0 489 612 640 646 650 654 656 656 656 656
–50% ≤ VRR < 0 0 214 255 261 264 266 267 268 268 268 268
VRR < –50% 0 194 210 212 216 219 219 219 219 220 220
Time(Days)

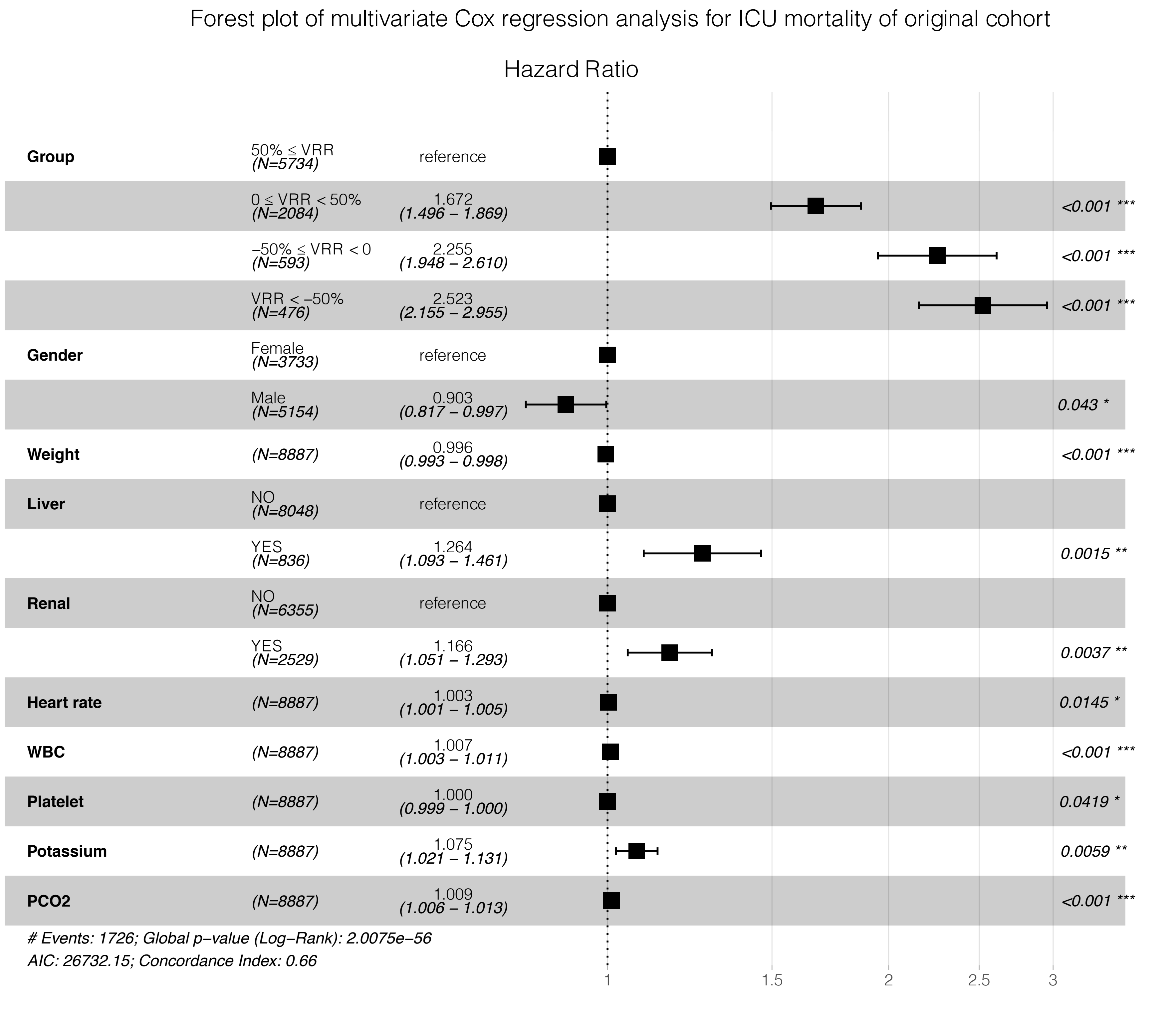
Forest plot of multivariate Cox regression analysis for ICU mortality of original cohort
Hazard Ratio
Group
50% ≤ VRR (N=5734) reference
0 ≤ VRR < 50% (N=2084) 1.672 (1.496 – 1.869) <0.001 ***
–50% ≤ VRR < 0 (N=593) 2.255 (1.948 – 2.610) <0.001 ***
VRR < –50% (N=476) 2.523 (2.155 – 2.955) <0.001 ***
Gender
Female (N=3733) reference
Male (N=5154) 0.903 (0.817 – 0.997) 0.043 *
Weight (N=8887) 0.996 (0.993 – 0.998) <0.001 ***
Liver
NO (N=8048) reference
YES (N=836) 1.264 (1.093 – 1.461) 0.0015 **
Renal
NO (N=6355) reference
YES (N=2529) 1.166 (1.051 – 1.293) 0.0037 **
Heart rate (N=8887) 1.003 (1.001 – 1.005) 0.0145 *
WBC (N=8887) 1.007 (1.003 – 1.011) <0.001 ***
Platelet (N=8887) 1.000 (0.999 – 1.000) 0.0419 *
Potassium (N=8887) 1.075 (1.021 – 1.131) 0.0059 **
PCO2 (N=8887) 1.009 (1.006 – 1.013) <0.001 ***
Events: 1726; Global p-value (Log-Rank): 2.0075e-56
AIC: 26732.15; Concordance Index: 0.66
1
1.5
2
2.5
3
