# Supplementary material for: Tendency of dynamic vasoactive and inotropic medications data as a robust predictor of mortality in patients with septic shock: An analysis of the MIMIC-IV database
Source: Front Cardiovasc Med. 2023 Mar 7;10:1126888. doi: 10.3389/fcvm.2023.1126888 (PMC10112491; doi:10.3389/fcvm.2023.1126888)
Supplement: Supplementary file 4 [file Image_3.PDF]

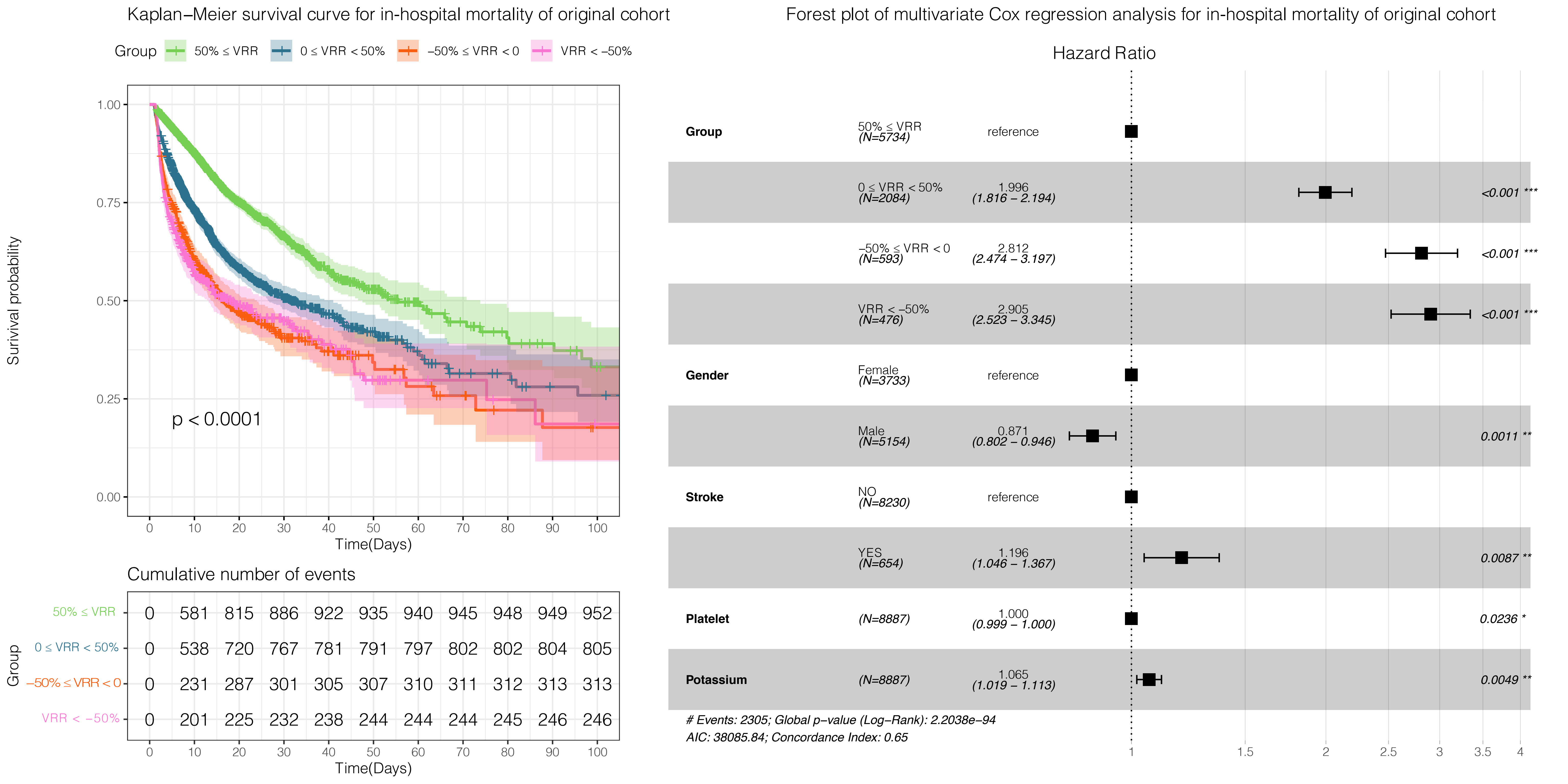

Kaplan–Meier survival curve for in-hospital mortality of original cohort
Group
50% ≤ VRR
0 ≤ VRR < 50%
–50% ≤ VRR < 0
VRR < –50%
Surivival probability
1.00
0.75
0.50
0.25
0.00
p < 0.0001
0 10 20 30 40 50 60 70 80 90 100
Time(Days)
Cumulative number of events
Group
50% ≤ VRR 0 581 815 886 922 935 940 945 948 949 952
0 ≤ VRR < 50% 0 538 720 767 781 791 797 802 802 804 805
–50% ≤ VRR < 0 0 231 287 301 305 307 310 311 312 313 313
VRR < –50% 0 201 225 232 238 244 244 244 245 246 246
0 10 20 30 40 50 60 70 80 90 100
Time(Days)
Forest plot of multivariate Cox regression analysis for in-hospital mortality of original cohort
Hazard Ratio
Group 50% ≤ VRR (N=5734) reference
0 ≤ VRR < 50% (N=2084) 1.996 (1.816 – 2.194) <0.001 ***
–50% ≤ VRR < 0 (N=593) 2.812 (2.474 – 3.197) <0.001 ***
VRR < –50% (N=476) 2.905 (2.523 – 3.345) <0.001 ***
Gender Female (N=3733) reference
Male (N=5154) 0.871 (0.802 – 0.946) 0.0011 **
Stroke NO (N=8230) reference
YES (N=654) 1.196 (1.046 – 1.367) 0.0087 **
Platelet (N=8887) 1.000 (0.999 – 1.000) 0.0236 *
Potassium (N=8887) 1.065 (1.019 – 1.113) 0.0049 **
Events: 2305; Global p-value (Log-Rank): 2.2038e-94
AIC: 38085.84; Concordance Index: 0.65
1 1.5 2 2.5 3 3.5 4
